# Comparative genomics revealed the gene evolution and functional divergence of magnesium transporter families in *Saccharum*

**DOI:** 10.1186/s12864-019-5437-3

**Published:** 2019-01-24

**Authors:** Yongjun Wang, Xiuting Hua, Jingsheng Xu, Zhichang Chen, Tianqu Fan, Zhaohui Zeng, Hengbo Wang, Ai-Ling Hour, Qingyi Yu, Ray Ming, Jisen Zhang

**Affiliations:** 1 Center for Genomics and Biotechnology, Haixia Institute of Science and Technology, Fujian Provincial Key Laboratory of Haixia Applied Plant Systems Biology, College of Resources and Environment, College of Life Sciences, Fujian Agriculture and Forestry University, Fuzhou, 350002 China; 2 Guangxi Key Lab of Sugarcane Biology, Guangxi University, Nanning, Guangxi China; 3 Root Biology Center, Fujian Agriculture and Forestry University, Fuzhou, 350002 Fujian China; 4 Department of Life Science, Fu-Jen Catholic University, Xinzhuang Dist., Taibei, 242 Taiwan; 5 Texas A&M AgriLife Research, Department of Plant Pathology and Microbiology, Texas A&M University System, Dallas, TX 75252 USA; 6 Department of Plant Biology, University of Illinois at Urbana-Champaign, Urbana, IL 61801 USA

**Keywords:** *Saccharum*, Magnesium transporter, Gene evolution, Gene expression

## Abstract

**Background:**

Sugarcane served as the model plant for discovery of the C_4_ photosynthetic pathway. Magnesium is the central atom of chlorophyll, and thus is considered as a critical nutrient for plant development and photosynthesis. In plants, the magnesium transporter (*MGT*) family is composed of a number of membrane proteins, which play crucial roles in maintaining Mg homeostasis. However, to date there is no information available on the genomics of *MGTs* in sugarcane due to the complexity of the *Saccharum* genome.

**Results:**

Here, we identified 10 *MGTs* from the *Saccharum spontaneum* genome. Phylogenetic analysis of *MGTs* suggested that the *MGTs* contained at least 5 last common ancestors before the origin of angiosperms. Gene structure analysis suggested that *MGTs* family of dicotyledon may be accompanied by intron loss and pseudoexon phenomena during evolution. The pairwise synonymous substitution rates corresponding to a divergence time ranged from 142.3 to 236.6 Mya, demonstrating that the *MGTs* are an ancient gene family in plants. Both the phylogeny and Ks analyses indicated that *SsMGT1/SsMGT2* originated from the recent ρWGD, and *SsMGT7/SsMGT8* originated from the recent σ WGD. These 4 recently duplicated genes were shown low expression levels and assumed to be functionally redundant. *MGT6, MGT9* and *MGT10* weredominant genes in the *MGT* family and werepredicted to be located inthe chloroplast*.* Of the 3 dominant *MGTs, SsMGT6* expression levels were found to be induced in the light period*,* while *SsMGT9* and *SsMTG10* displayed high expression levels in the dark period. These results suggested that *SsMGT6* may have a function complementary to *SsMGT9* and *SsMTG10* that follows thecircadian clock for MGT in the leaf tissues of *S. spontaneum*. *MGT3, MGT7* and *MGT10* had higher expression levels In*saccharum officinarum* than in *S. spontaneum*, suggesting their functional divergence after the split of *S. spontaneum* and *S. officinarum*.

**Conclusions:**

This study of gene evolution and expression of *MGTs* in *S. spontaneum* provided basis for the comprehensive genomic study of the entire *MGT* genes family in *Saccharum*. The results are valuable for further functional analyses of *MGT* genes and utilization of the MGTs for *Saccharum* genetic improvement.

**Electronic supplementary material:**

The online version of this article (10.1186/s12864-019-5437-3) contains supplementary material, which is available to authorized users.

## Background

Magnesium (Mg) is the 8th most abundant element on earth, the 4th most abundant element in vertebrates, and the 2nd most abundant cation in plants. Mg’s ionic radius is among the smallest but the hydrated radius is so far the largest of all cations [1]. Due to its unique chemical property, Mg plays an essential role in plant growth and development, such as being the central atom of the porphyrin ring of chlorophyll, enabling plants to perform photosynthesis [2, 3], and a co-enzyme in the form of Mg^2+^-ATP complexes or acts as a cofactors for more than 300 enzymes that are involved in enzyme activation [4–6]. Mg also alleviates plant toxicity from aluminum (Al) and heavy metals [7], and is involved in protein synthesis by bridging ribosome sub-units [8]. Due to its potential for leaching in highly weathered soils and the interaction with Al [9], Mg^2+^ bind weakly to negative charged soil colloids and root cell walls by its unique chemical property, which leads to the easy loss of exchangeable Mg^2+^ from soil. As a result, the magnesium deficiency is an issue of crucial importance in acid soils [10]. One third of the tropics, or 1.7 billion hectares is acid soil [11]. Consequently, the magnesium deficiency hindered the production of tropical crop such as sugarcane.

Since Mg is one of the essential macroelements in plants, deficiency of Mg causes marked inhibition of plant growth and development, including symptoms such as leaf interveinal chlorosis, particularly in mature leaves due to the high mobility of Mg in plants [9], which therefore decreases photosynthesis efficiency. Mg deficiency alsonegatively impacts carbon allocation to sink organs [12], and inhibits plant root growth [13], which leads to a deleterious effect on both crop productivity and quality [12].

Plant Mg-uptake from soil solution is controlled by 2 predominantly processes: mass flow and diffusion. To maintain concentration of Mg in tissues, plants have a series of highly-efficiency transport methods for Mg uptake, storage and translocation. The MGT family is composed of several membrane proteins that maintain Mg homeostasis to support plant growth. The main *MGT* genes were originally identified from *Salmonella typhimurium*, which belongs to the CorA family of bacteria [14]. In *Salmonella typhimurium*, CorA is a single copy gene encoding a 37-kD integral membrane protein [15]. In plants, Li et al. (2001) first identified 10 MGTs in the model plant *Arabidopsisthaliana* [16]. MGT families were also identified in rice (*Oryza sativa*) [17] and maize (*Zea mays*) [18], However, the relationships between gene structure and gene divergence of *MGTs* have not been reported in previous studies. Gene families were originated from duplicated events including the whole genome duplication (WGD), segment duplication and tandem duplication, playing key roles in organism evolutionary process. The retained duplicated genes tend to diverge in regulatory and coding regions and were accomplished by three main types of mechanisms, exon/intron gain/loss, exonization/pseudoexonization, and insertion/deletion [19]. In *Arabidopsis*, *AtMGT9* was found to play an essential role in pollen development [20], and *AtMGT10* is essential for chloroplast development and photosynthesis [21], whereas *AtMGT6,* as a Mg uptake transporter, is required for growth under low Mg conditions [22]. In *Oryza sativa*, *OsMGT1* was found to play an important role in the tolerance to Al toxicity and salt stress [23]. In maize, *ZmMGT10* is essential in response to Mg deficiency, and confers low Mg tolerance when the *ZmMGT10* gene is overexpressed in *Arabidopsis thaliana* [24].

Identifying MGTs in sugarcane and studying the regulatory mechanism in response to Mg stress is of particular interest because sugarcane is a model C_4_ crop. Indeed, sugarcane contributes about 80% of the world sugar and about 40% of ethanol production worldwide. Consequently, the effects of Mg on sugarcane yield and quality have been studied, and field experiments have clearly established that yield can be significantly improved when N and P utilization are balanced with K, S, and Mg. [25, 26]. However, these studies largely focused on phenotypic features, physiological function and the pathology of Mg deficiency [12, 27–29]. In addition, due to hyper-ploidy, the modern sugarcane cultivar has one of the most complex genomes among all the crops, being both an aneuploid and autopolyploid with an extreme ploidy level that can range from octoploidy (x = 8) to dodecaploidy (x = 12). The chromosome number of different species in the genus *Saccharum* ranges from 36 to 170 (*S. officinarum*, 2n = 70 to 140; *S. robustum*, 2n = 66 to 170; *S. spontaneum*, 2n = 36 to 128) [30]. To date, our understanding of the regulatory mechanism in response to Mg stress and MGT in *Saccharum* remains very limited due to lack of reference genome in the past.

In this study, basing on the recent sequencing *S. spontaneum* genome [31], we identified the MGTs in *S. spontaneum* (*SsMGTs*). Phylogenetics, comparative genomics and gene expression patterns were used to study the evolutionary relationship and potential function of MGT families in *Saccharum*.

## Results

### Identification of *MRS2/MGT* family genes in *S. spontaneum*

Based on comparative genomics technology, ten members of *MRS2/MGTs* were identified from the *Sorghum bicolor* genome (Table 1). Using these sorghum *MGTs* as a reference, 10 orthologous *MGTs* were identified in the monoploidy genome database of AP85–441 [31]. Open reading frame of the *SsMRS2/MGTs* were analyzed based on an online tool ORF finder [32]. The SsMRS2/MGT proteins were 290–539 amino acid (aa) residues in length, with an average length of 414 aa, and the corresponding molecular masses ranged from 39.13 to 58.68 kDa, average molecular weight was 47.63 kDa, the computed theoretical isoelectric points varied greatly, ranging from 4.66(*SsMGT1*) to 9.29(*SsMGT5*). These 10 genes were named *SsMGT1*-*SsMGT10*.

**Table 1:** Information about the putative *MGT* genes in *Sorghum bicolor*

Corresponding genename in sugarcane	GeneID	TranscriptID	ProteinID	Gene location
*SsMGT1*	Sobic.006G082200	XM_002446449	XP_002446494	Chr06:45079489..45081310forward
*SsMGT2*	Sobic.007G185200	XM_002445725	XP_002445770	Chr07:61831349..61833678reverse
*SsMGT3*	Sobic.003G395600	XM_002458906	XP_002458951	Chr03:70601663..70609036 reverse
*SsMGT4*	Sobic.006G137200	XM_002446702	XP_002446747	Chr06:49948483..49953310 forward
*SsMGT5*	Sobic.010G210500	XM_002438717	XP_002438762	Chr10:55405378..55409126 reverse
*SsMGT6*	Sobic.001G095000	XM_002463854	XP_002463899	Chr01:7305915..7311750 forward
*SsMGT7*	Sobic.001G309400	XM_002464953	XP_002464998	Chr01:59477626..59480767 forward
*SsMGT8*	Sobic.001G512100	XM_002465855	XP_002465900	Chr01:78001611..78004021 forward
*SsMGT9*	Sobic.001G135100	XM_002466617	XP_002466662	Chr01:10653760..10659627 reverse
*SsMGT10*	Sobic.003G395600	XM_002458906	XP_002458951	Chr03:70601663..70609036 reverse

These sorghum *MGTs* were also used as reference to search the recent published genome of *Saccharum* hybridR570 [33], and 9 *MGTs* were identified (Additional file 1). Noteworthy, a pair of tandem duplicated genes (*Sh06 p004330* and *Sh06 p004340*) were found in the *Saccharum* hybridR570 genome, and were likely originated from *S. officinarum* as the tandem duplicated genes of *MGT* which were not found in *S. spontaneum* (Additional file 2). These two *ShMGTs* shared high (72%) sequence similarity, suggesting that they were originated from the very recent tandem duplication event. Two orthologs of *SsMGTs* (*SsMGT3, SsMGT6*) were absented in the R570 genome, which may be caused by the incomplete *Saccharum* genome (Additional file 1).

**Fig. 1:**
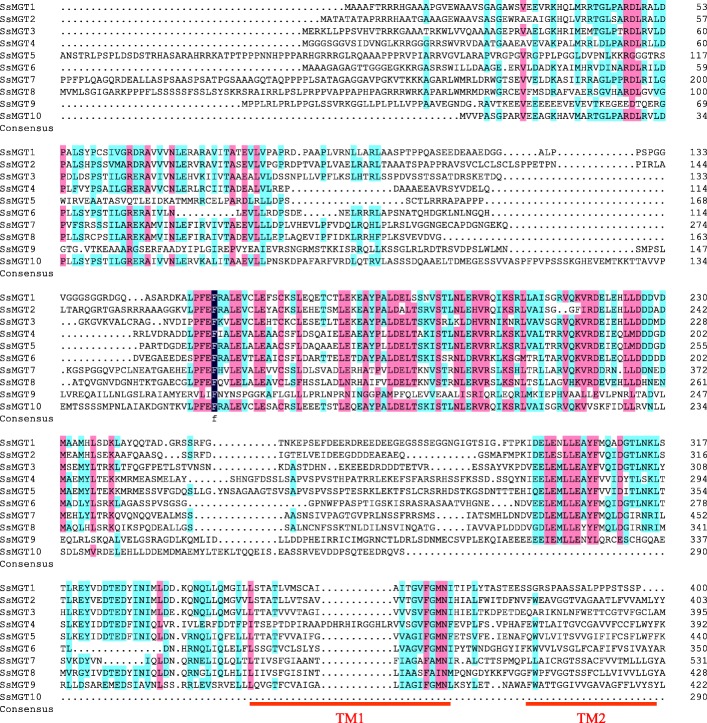
Multiple protein sequence alignment of the *SsMGT* family was performed using by DNAMAN software. The degree of similarity of 10 *SsMGTs* protein sequences are displayed in different colors (100%, deep blue; 75–99%, cherry red and 50–74% colored by cyan). TM domains are marked with red lines, the typical conserved GMN motifs are highlighted by red boxes

A multiple protein sequence alignment was performed to analyze the conservation of motifs in the examined plant species (Fig. 1). Eight of the*SsMGTs* in sugarcane shared a common GMN motif with *Arabidopsis*, *Oryza sativa* and *Zea mays* except for Ss*MGT*7 and *SsMGT8*. Ss*MGT*7 contained an AMN motif instead of GMN, and *SsMGT8* contained an AIN motif instead of GMN, similarly to *OsMRS2/MGT* in *Oryza sativa* [17]. Nine of these *SsMRS2/MGT* contained the complement CorA TM domain except for *SsMGT10* [34].

Using the WoLF PSORT program, 8 of the SsMGT proteins were predicted to be located in chloroplasts, the remaining 2 SsMGT proteins were predicted to be localized in the mitochondria (SsMGT3) or plasma membrane (SsMGT5) (Table 2), indicating that these SsMGTs mainly play a role in the chloroplasts to maintain Mg^2+^ homeostasis in sugarcane. The analysis of the TM domain revealed that 9 of the SsMGTs, except SsMGT10, contained 9 protein TM domains near their C-terminal ends, which were similar to the CorA/MRS2/MGT transporters from other plant species, including *Arabidopsis*, rice and maize. Significantly, both SsMGT1 and SsMGT3 contained only onetransmembrane domain, and SsMGT10 have no transmembrane domains (Additional file 3). Pairwise comparisons among the SsMGTs showed that all these MGTs are independent, and shared protein sequence similarities that ranged from 21 to 63% with an average of 39%, indicating theMGTs are an ancient gene family with high sequence divergence (Additional file 4).

**Table 2:** Characterization comparison of the *MGT* genes between *Sorghum bicolor* and *Saccharum*

*S. bicolor*								*S. spontaneum*								
Gene name	Gene ID	Amino acids size	Molecular weights(kDa)	Domains	Isoelectric point (PI)	Transmembrane helices	Predicted location^a^	Gene name	Gene ID	Amino acids size	Molecular weights(kDa)	Domains	Isoelectric point (PI)	Transmembrane helices	Identity%	Predicted location^a^
*SbMGT1*	Sobic.006G082200	421	45.02	Mrs2_Mfm1p-like	4.66	2	chlo	*SsMGT1*	AP850373380	400	42.92	Mrs2_Mfm1p-like	4.66	1	89.87%	chlo
*SbMGT2*	Sobic.007G185200	359	39.02	Mrs2_Mfm1p-like	4.82	2	chlo	*SsMGT2*	AP851129300	410	44.5	Mrs2_Mfm1p-like	5.1	2	72.60%	chlo
*SbMGT3*	Sobic.003G369600	414	46.21	Mrs2_Mfm1p-like	4.91	2	mito	*SsMGT3*	AP851069690	410	46.02	Mrs2_Mfm1p-like	5.15	2	93.25%	mito
*SbMGT4*	Sobic.006G137200	441	48.91	Mrs2_Mfm1p-like	5.27	2	plas	*SsMGT4*	AP850864340	399	45.07	Mrs2_Mfm1p-like	5.27	1	86.33%	chlo
*SbMGT5*	Sobic.010G210500	348	39.62	Mrs2_Mfm1p-like	4.82	2	plas	*SsMGT5*	AP851097490	446	49.75	Mrs2_Mfm1p-like	9.29	2	83.62%	Plas
*SbMGT6*	Sobic.001G095000	387	42.71	Mrs2_Mfm1p-like	4.86	2	cyto	*SsMGT6*	AP850291960	357	39.13	Mrs2_Mfm1p-like	5.09	2	89.72%	chlo
*SbMGT7*	Sobic.001G309400	478	52.38	Mrs2_Mfm1p-like	8.33	2	mito	*SsMGT7*	AP850490950	539	58.68	Mrs2_Mfm1p-like	7.7	2	91.49%	chlo
*SbMGT8*	Sobic.001G512100	432	48.3	Mrs2_Mfm1p-like	9.33	2	chlo	*SsMGT8*	AP851016400	492	55.02	Mrs2_Mfm1p-like	8.99	2	92.89%	chlo
*SbMGT9*	Sobic.001G135100	458	50.74	Mrs2_Mfm1p-like	5.38	2	chlo	*SsMGT9*	AP851096250	428	47.62	Mrs2_Mfm1p-like	5.3	2	97.19%	chlo
*SbMGT10*	Sobic.003G395600	443	48.55	Mrs2_Mfm1p-like	4.81	2	chlo	*SsMGT10*	AP851092670	290	31.91	Mrs2_Mfm1p-like	4.8	0	90.59%	chlo

^a^*chlo* Chloroplast, *mito* Mitochondria, *plas* Plasma membrane, *cyto* Cytoplastic

**Fig. 2:**
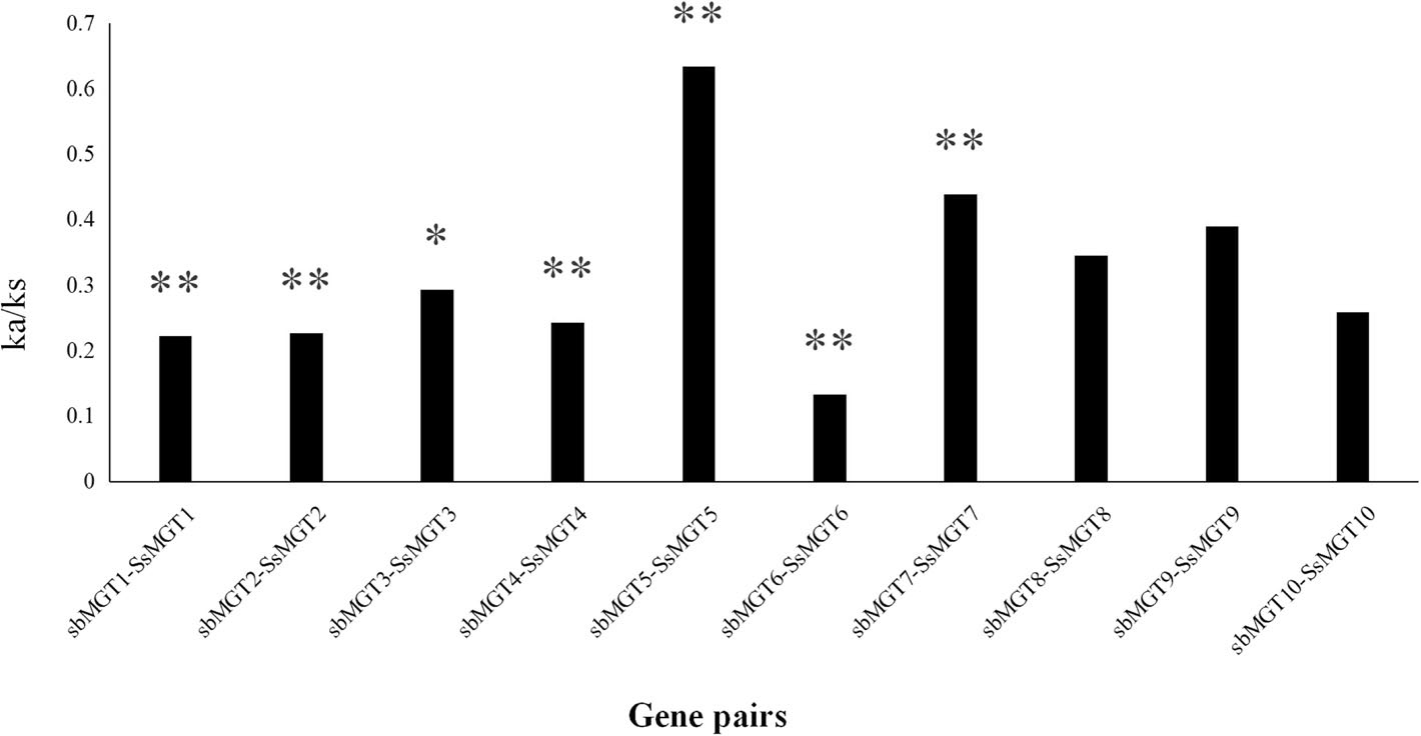
The non-synonymous (Ka) and synonymous (Ks) substitution ratios of the 10 pairs of orthologous *MGTs* genes from sorghum and sugarcane were calculated by the easy_kaks calculate program. * and ** represent *p* value< 0.05 and *p* value< 0.01 respectively

Nonsynonymous to synonymous substitution ratio (Ka/Ks) was analysed to investigate the evolutionary function constraint in *S. spontaneum*, 10 pairs of orthologous *SsMGT* genes between *S. spontaneum* and sorghum were calculated. The results show that, except for *SsMGT5*, the Ka/Ks ratios of other 9 genes were less than 0.5 (Fig. 2), suggesting that purifying selection was the main force for driving the evolution of *MGT* genes [35].

### Phylogenetic analysis of *SsMGTs* and other plant *MGTs*

**Fig. 3:**
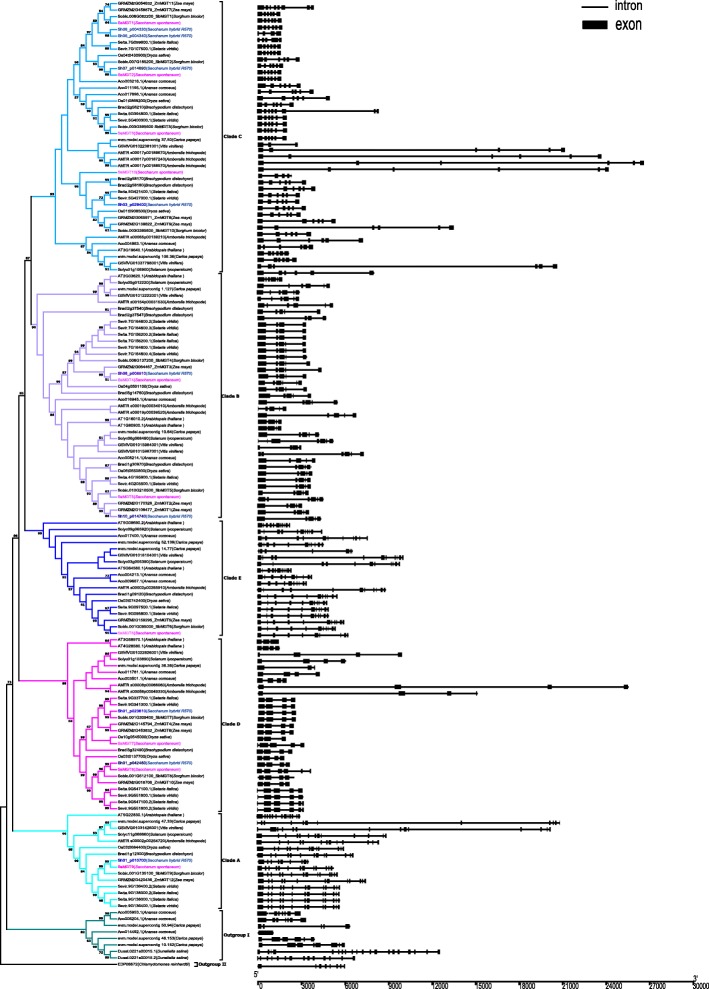
Phylogeny and schematic diagram for intron/exon organization of *MGT* genes from 14 plant species

**Fig. 4:**
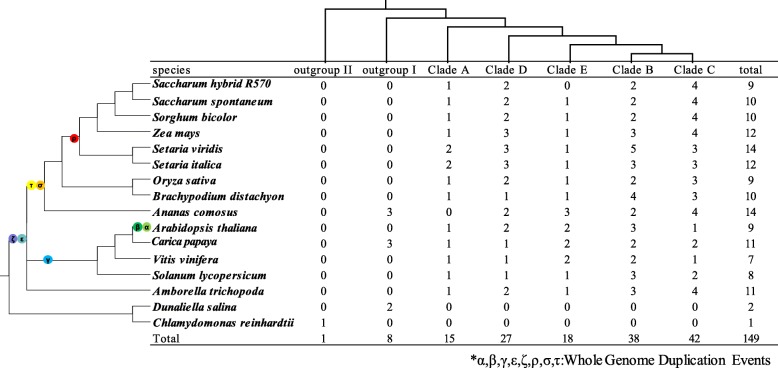
Phylogenetic relationships of *MGTs* families based on the current data for angiosperms

To analyze the phylogenetics of the *MGT* gene family, a total 149 *MGTs* from 16 representative plant species and 2 species as outgroups (*Chlamydomonas reinhardtii*and*dunaliella salina)* were used to construct the phylogenetic tree using the Neighbor-Joining and Maximum Likelihood method (Fig. 3, Additional files 5, 6 and 7 ). These *MGT* genes were phylogenetically divided into 5 clades (A, B, C, D and E) based on the previously reported *ZmMGTs* [18] (Additional file 7). The distribution of *MGT* gene numbers of the examined species showed that the ancient WGD did not contribute to the expansion of the *MGT* gene family (Fig. 4), whereas the MGT number in each clade gradually increased.Noteworthy, pineapple *MGTs* were divided into the outgroup, close to the *Chlamydomonas reinhardtii* and before *MGTs* of *Dunaliella salina*, speculating that horizontal gene transfer may contribute to the gene expansion of *MGT* in pineapple. Different from *ZmMGTs* research [18], phylogenetic tree in this study showed that the Clade D and Clade E were classed into different cluster, which may be caused by integrating more MGT families from representative species for phylogenetic analysis. Moreover, all 5 clades contained *MGT* genes from the 12 representative angiosperms plants, suggesting that *MGT* genes from each of the 5 clades appeared before the emergence of angiosperms. The evolution history of *MGT* clade could be sorted by age in duplicated descending order: Clade A, Clade D, Clade E, Clade B and Clade C.

Among the ten *SsMGT*genes, within clade A, *SsMGT9* was retained from the first round of gene duplication in angiosperms; within clade D, *SsMGT7* and *SsMGT8* were originated from last common ancestral (LCA) in the second round of gene duplication in angiosperms by a gene duplication event which occurred after the divergence of monocots and dicots; within clade E, *SsMGT6* was retained from the third round of gene duplication in angiosperms; within clade B, *SsMGT4* and *SsMGT5* were duplicated from LCA in the 4th round of gene duplication in angiosperms before the split of dicots and monocots; within clade C, *SsMGT1* and *SsMGT2* were derived from the ρWGD events, *SsMGT3* originated from the LCA of angiosperms, *SsMGT10* was retained in monocots from the gene duplication event before emergence of angiosperms.

### Exon/intron organization of the *SsMGTs* family and *MGTs* in other plant species

In the examined plant species, the gene structure of *MGTs* varied in both exon number and size. To investigate the structural characteristics and evolution of the *MGT* genes in different species, we analysed the gene features and patterns of the *MGTs* (Fig. 3). The exon number of *MGTs* ranged from 1 to 20 with an average about 7 (Additional files 8 and 9), and 103 of the 149 *MGTs* have exon numbers ranging from 4 to 6. We thus speculated that the LCA exon number of angiosperms *MGT* gene is between 4 and 6. Each of the subgroups had a similar exon number except for *Ananas comosus*.

The exon number of *SsMGTs* ranged from 5 to 13, and 6 of *SsMGTs* harboured 6 exons (Additional file 8). Moreover, the *SsMGTs* gene structure showed a pattern similar to *SbMGTs* and *ZmMGTs* from the same clade, suggesting the conservatism of the *MGT* gene structure in the *Panicoideae*. In clade A, *MGTs* genes have higher exon number than in other clades. These genes contained 9–14 exons of *MGTs* genes, which is likely due to the exon splitting as their protein sizes remained consistent. In clade B, *MGTs* exon number ranged from 4 to 7, The first exon and second intron of *SsMGT5 is* larger than that of *SbMGT5*, which indicated an expansion and exonization of the second intron. In clade C, *MGTs* exon number ranged from 4 to 8, while the number of *SsMGT* exons was conserved. In clade D, *MGTs* exon number ranged from 3 to 6, gene structures were more conserved in monocots than in dicots, and gene expansions only existed in the dicots. Important to note, both *SsMGT7 and 8* have one more exon than the other monocots *MGTs*, which was likely caused by exonization. In clade E, *MGTs* exon number ranged from 7 to 12, the exon number of *SsMGT6* was less than the other *MGTs,* which may be caused by the pseudo-exonization in this gene. These results suggested that *MGTs* experienced gene structure reconstruction under different evolutionary dynamics in *S. spontaneum* as well as in angiosperms.

Based on the pairwise synonymous substitution rates (Ks) in angiosperms (Table 3, Additional file 10), we estimated the divergence time among 5 clades of *MGT* family. The median values of pairwise Ks ranged from 1.74 to 2.89, corresponding to a divergence time ranging from 142.3 to 236.6 Mya, indicating that the *MGTs* in the 5 clades are ancient and divergent. Moreover, the divergence times among the 4 *SsMGTs* ranged from 52.6 to 96.7 Mya (Table 4). Taken together, these results suggest that the MGT family is an ancient gene family with a recent gene duplication event in *Gramineae*.

**Table 3:** Divergence time among 5 clades of *MGTs* family in angiosperms

Clade-Clade	Median Ks	Gene pairs used	Divergence time (Mya)
A-B	2.88637	234	236.6
A-C	2.49044	162	204.1
A-D	2.30398	270	188.9
A-E	2.16215	172	177.2
B-C	1.73644	412	142.3
B-D	1.9539	460	160.2
B-E	2.20187	436	180.5
C-D	2.29602	608	188.2
C-E	2.26799	355	185.9
D-E	2.47121	252	202.6

**Table 4:** Divergence between paralogous *SsMGTs* pairs in*S. spontaneum*

Gene1	Gene2	Ka	Ks	Ka/Ks	Divergence time (Mya)	*P*-Value (Fisher)
SsMGT1	SsMGT2	0.268	0.642	0.417	52.6	1.56519E-11
SsMGT7	SsMGT8	0.347	1.180	0.295	96.7	6.86283E-24

### Gene expression and functional analysis of *MGTs* in two *Saccharum* species

Gene expression pattern information provides initial reference for evaluating their potential function in plant. In this study, to understand the potential function of *MGTs* in *Saccharum* species, we investigated gene expression patterns based on 4 sets of RNAseq data: 1) Different developmental stages;2) Leaf gradient; 3) Time course and 4) Hormone treatment. The Reads Per Kilobase of exon per Million (RPKM) reads of*MGT6*, *MGT9* and *MGT10* in each leaf segment (LF1, 6, 10, and 15) were further verified by qRT-PCR data. The results were positively correlated with RPKM values (Additional files 11 and 12), demonstrating the reliability of gene expression results based on RNA-seq analysis.

**Fig. 5:**
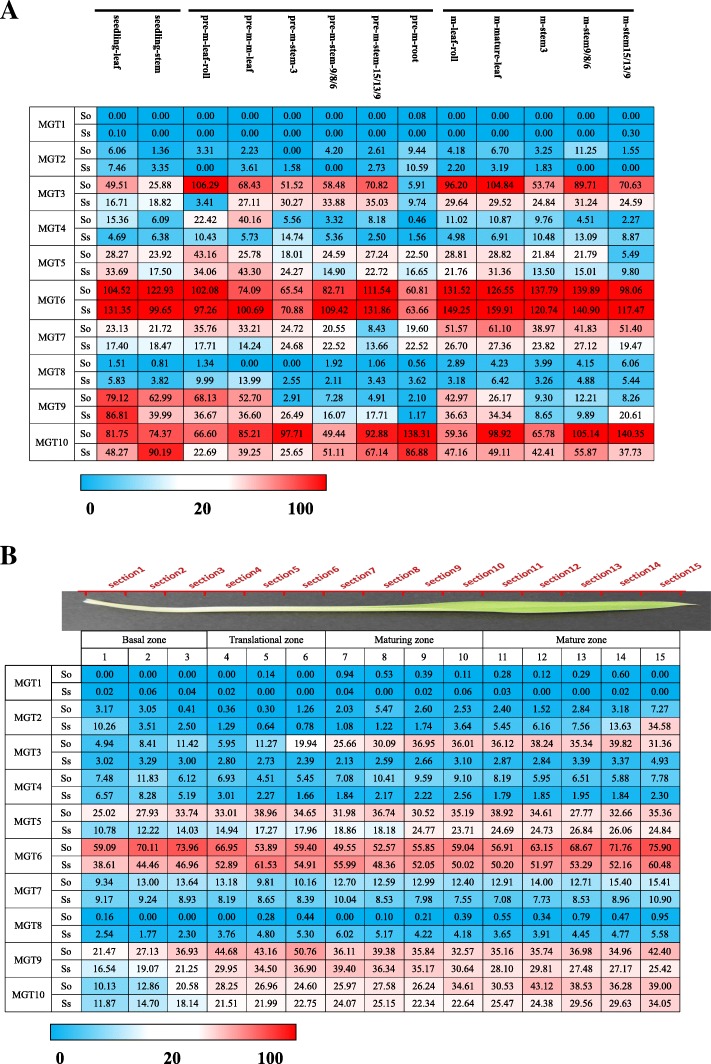
**a** The expression pattern of *MGTs* based on RPKM in 2 *Saccharum* species. **b** The expression patterns of *MGTs* based on RPKM across leaf segments in 2 sugarcane species

In the *SaccharumMGT* families, transcription of *MGT1* was undetectable in all the examined tissues from *Sacchaurm* plants.*MGT2/4/5/8* presented low expression in all the examined tissue (Fig. 5a).*MGT*6, *MGT9* and*MGT10* had predominant expression levels among the gene families, indicating that the 3 genes were the main members in the *MGT* families.*MGT3/7/10* showed varying expression levels in the examined tissues from the 2 *Saccharum* species.

### Expression pattern of *MGTs* at different developmental stages

To investigate gene functional divergence among the *Saccharum* species, we analyzed the transcriptome profile of *MGTs* among 2 *Saccharum* species, *S. officinarum* and *S. spontaneum,* at 3 developmental stages and in 6 different tissues, including the 2 leaf (leaf, leaf roll) and 3 stalks (mature, maturing and immature) and the root tissue of pre-mature *Saccharum* (Fig. 5a, Additional file 13). Among the 10 *MGTs* analyzed, 4 genes (*MGT1, MGT2, MGT4* and *MGT8*) displayed very low or undetectable levels in all the examined tissues. *MGT3* presented higher expression levels in leaf and mature stem (sink tissues) at the mature stage than the young tissues, higher in *S. officinarum* than in the *S. spontaneum*, and higher in the leaf than in the stem. *MGT6* was the most highly expressed gene among the gene families and displayed a constitutive expression pattern.Both *MGT5* and *MGT9* were observed to have higher expression levels in leaf tissues including the mature leaf and leaf roll than in the stems and root. In leaf tissue, *MGT9* expression levels were observed to decrease gradually during the development of the *Saccharum*species, and *MGT9* likely functions in the seedling period of sugarcane development. In contrast to *MGT5* and *MGT9, MGT10* was abundant in the examined tissues and presented higher expression levels in mature stems than in other tissues.

### Expression pattern of MGT under hormone treatment

Phytohormone-mediated regulation of gene expression plays the crucial role in plant growth and development. After subjected to magnesium deficiency and magnesium toxicity stress, abscisic acid (ABA), ethylene and other signal transduction related hormone concentrations and its transporter’s expression levels in *Arabidopsis thaliana* were significantly up-regulated or down-regulated [36–38]. In this research, we analyzed the gene expression in the leaves of seedlings of *Saccharum* plants treated with ABA and GA. Gene expression levels were altered in response to ABA or GA treatment, especially for *MGT3*, *MGT4*, *MGT9* and *MGT10* (Additional file 14). Furthermore, the gene expression of different *MGTs* varied in response to different ABA treatment times. For instance, *SoMGT3* and *SoMGT9* showed a rise sharply in leaf, whereas *SsMGT4* in leave, *SoMGT8*, *SoMGT9*, *SsMGT10* and *SoMGT10* in stem were decreased its expression level as the ABA treatment time was increased. Under GA treatment, the expression of *SoMGT2*, *SoMGT3* and *SsMGT9* was induced in leaf, the transcript of *SoMGT4* and *SoMGT5* wasinduced in the stem tissue. The expression of *MGT10* was suppressed in the stem tissue. The transcript of *SsMGT4* was inhibited in the leaf tissue at 48 h and 96 h, but was induced in the stem tissues at 96 h.

### Expression pattern of *MGTs* during the circadian rhythms

**Fig. 6:**
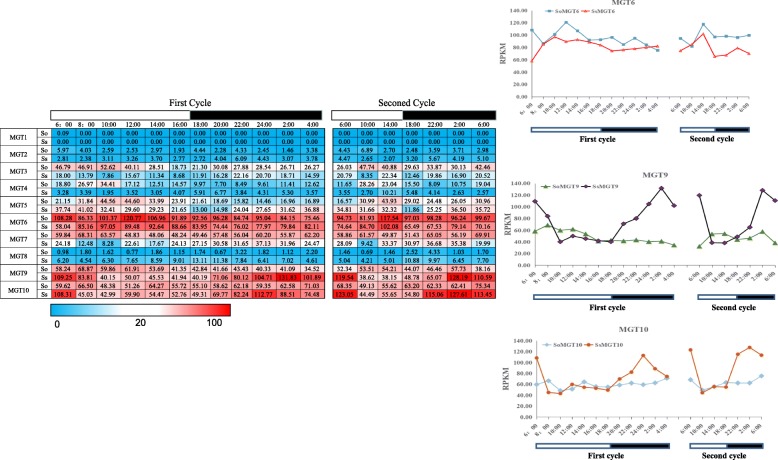
The expression pattern of *MGTs* based on RPKM in circadian rhythms

Since Mg is the central element in chlorophyll [3] and the plantsphotosynthetic apparatus is regulated by the circadian clock at the transcriptional level [39], we performed gene expression analysis for *MGT* during the diurnal cycles in the 2 *Saccharum* species.*MGT9* and *MG10* were observed to have a peak expression in the middle of the night period in *S. spontaneum*, but showed no diurnal expression in *S. officinarum*, indicating diurnal rhythms regulate these 2 *MGTs* in *S. spontaneum* rather than *S. officinarum* (Fig. 6). In contrast, the dominant *MGT6* displayed a diurnal expression pattern with peak levels in the middle of the day in both of the 2 analyzed *Saccharum* species, suggesting *MGTs* correlate with diurnal rhythms in *Saccahrum* (Fig. 6).

### Expression pattern of *MGTs* in different developmental gradient of the leaf

To further understand the functional divergence for photosynthesis, we exploited the continuous developmental gradient of the leaf transcriptome of *MGTs* in maize, rice and  2 *Saccharum* species (Fig. 5b, Additional file 15). Consistently, 4 genes (*MGT1, MGT2, MGT4* and *MGT8*) displayed very low or undetectable expression levels in all the examined segments of the leaf tissues (Fig. 5b). The expression of *MGT3* and *MTG5* gradually increased from the base to the tip of the leaf in *S. officinarum* and showed higher expression levels in *S. officinarum* than in *S. sponteneum*, indicating gene functional divergence after the split of *S. spontaneum* and *S. officinarum*. *MGT6* presented abundant expression level in all the examined leaf segments, further supporting that this gene is aconstitutively expressed gene in the *MGT* family*. MGT9’s* expression was high in the translational zone but low in the basal zone. *MGT10* displayed gradually increase expression from the base to the tip of the leaf in both of the 2 analyzed *Saccharum* species.

### *SsMGT3* complements a *S. typhimurium* strain MM281 deficient in Mg^2+^ uptake

To verify the *SsMGTs* function for magnesium transportation, we transferred the *SsMGT3*to *Salmonellatyphimurium* strain MM281 which is lack of Mg-transporting systems [16, 40, 41]. The MM281 cells do not survive in the low-Mg medium (< 1 mM) at pH 7.5 [42], but the transformed MM281 could survive in N-minimal medium containing low-Mg concentration. The result show that *SsMGT3* could recover the magnesium uptake function in MM281 (Additional file 16). Supporting that *SsMGTs* play vital role in transporting magnesium in sugarcane.

## Discussion

Mg plays a fundamental role in plants since 15–20% of total Mg participates in chloroplast formation in the leaf. Due to its tendency to form octahedral complexes, Mg also plays a predominant role as a cofactor of a series of enzymes involved in photosynthetic carbon fixation and metabolism, with the remaining fraction being stored in the vacuole [43]. The *CorA* gene in bacteria, the *MRS2/MGT* gene in *Arabidopsis thaliana* and *Oryza sativa* participate in magnesium transport [16–18, 44]. Mg is typically highly mobile in plants [41]. The Mg uptaken by roots is uploaded to the xylem and transported from the root to the young leaf via long-distance transportation through stems. Due to its high mobility, Mg is also transported from old leaves to young leaves (i.e. transport from source to sink), and in a continuous supply cycle, as Mg is transported from the stem to the roots [45]. Ten members of *MGT* gene family were identified in *Saccharum* inthe monoploidy genome of AP85–441 [31]. Based on comparative genomics and gene expression patterns of the *MGT* families, gene evolution andtheir potential function divergence for photosynthesis wereinvestigated to further understand the mechanism of *MGT* in *Saccharum*.

WGD or polyploidization, gene deletion and diploidization, are the major driving forces for the divergence and biological diversity of angiosperms [46]. The commelinids originated from about 120 to 100 Mya, approaching to the origin of monocotyledons from about 150 to 130 [47]. Three ancient WGD events (τ, σ and ρ) occurred before the emergence of the Poaceae lineage. A recent study demonstrated that the number of WGD events in pineapple have one less ancient WGD (ρ) event than other sequenced gramineous plants [48]. In this study, we selected the *MRS2/MGTs* from 16 species including 14 representative plant species and 2 outgroups for phylogenetic analysis, providing the representative phylogenetic position for analyzing the gene evolution. And based on previous study about the WGD in angiosperms [48–52], along with the phylogeny relationship of *MGT* genes, *MGT* families in angiosperms have undergone over 4 rounds of gene duplications after the ε ancient WGD event. So, we put forward a hypothesis that the *MGT* is an ancient gene family with at least 5 LCAs prior to the emergence of angiosperms. The gene number of the 5 clades were similar among the 14 representative plant species despite the different ancient WGD events that occurred in these plant species, suggesting that gene expansion of *MGT* in angiosperm was not caused by ancient WGD events. It is obvious that gene loss occurred following the WGD events in the lineages of *A. thaliana* and lineages of Poaceae, which were mainly due to the gene functional redundancy of the *MGT* family as the multiple LCA existed before these WGD events. The divergence of 5 clades of the *MGT* gene family was estimated using the Ks in this study (Tables 3 and 4), and the results suggested that the *MGT* families in angiosperms (about 142~237 Mya) probably occurred before σ WGD in angiosperm (about 130 Mya) and after ε WGD in gymnosperm (about 245 Mya) [49].

By using more representative phylogenetic position for *MGT* family analysis, we were able to estimate the evolutionary history of the clades in duplicated descending order, clade A, clade D, clade E, clade B, and clade C. The results showed that the clade D was retained from the second gene duplication event, while clade E originated from the third gene duplication event, which is inconsistent with previous studies [17, 18]. However, the phylogenetic analysis for *MGT* from 2 previous studies only used 3 plant species (maize, rice and Arabidopsis) or 2 plant species (rice and Arabidopsis), making it possible that the reduced number of plant species provided insufficient information for phylogenetic construction and discrimination of clade position in the phylogenetic tree.

**Fig. 7:**
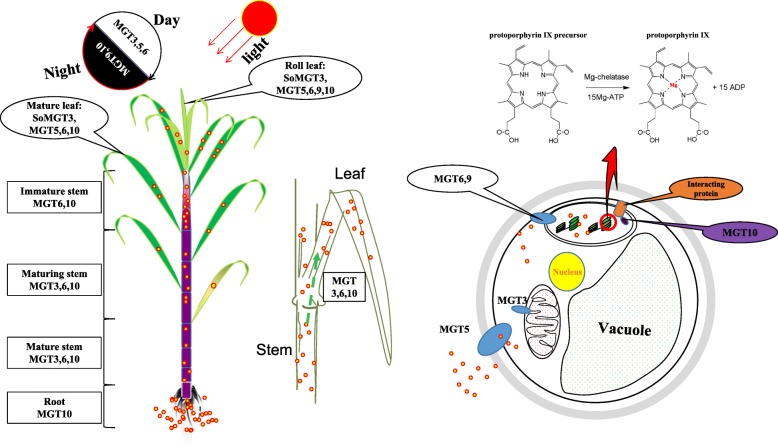
Schematic model of MGT proteins based on gene expressions in sugarcane. MGT3 presented higher expression levels in leaves and mature stems (sink tissues) at the mature stage than that in the young tissues, in *S.officinarum* than in the *S. spontaneum*, and in leaf than in the stem. *OsMGT1/OsMRS2–2* astheclosest orthologous genes of *SoMGT3* specifically response to aluminum stress and salt stress at the root [58], indicating that *SoMGT3* is likely to be involved in magnesium homeostasis in mature stems and leaf tissues during the light period in *S. officinarum,* but plays a limited role in *S. spontaneum*. Mg^2+^ is inserted into protoporphyrin IX via the magnesium-chelatase enzyme [2]. *MGT6* was the most highly expressed gene among the gene families and displayed a constitutive expression pattern. It was induced by light with an expressional peak at noon. In pre-mature plants, *MGT6* was highly expressed in the leaf and lowly expressed in roots. Magnesium concentrations of the chloroplast matrix in the light period were higher than in the dark period [90]. These results suggested that *MTG6* is the dominant *MGT* for the maintenance of magnesium concentration in chloroplast during the light period in *Saccharum*. *MGT5* and *MGT9* had higher expression level in leaf than in the stems and root. In leaf, expressional level of *MGT9* was gradient decrease from the base to the tip of leaf in the *Saccharum*, suggesting that *MGT5* and *MGT9* possibly played roles in leaf of sugarcane, and *MGT9* probably functioned in the seeding stage of sugarcane. While *MGT10* was abundant in the examined tissues and presented higher expression levels in root and mature stem than in other tissues, suggesting that *MGT10* probably played a complementary role in magnesium transport in leave tissues in the light and dark period in *S. spontaneum.* It also may be responsible for the long-distance transport of magnesium from source (root) to sink (leaf)

Distributed in clade A, *SsMGT9* was retained from the first round of gene duplication in angiosperms. It is interesting that except pineapple, all other plant species contained at least 1 *MGT* in Clade A. The Ka/Ks of *SsMGT9/SbMGT9* also supported SsMGT9 being under functional constraining divergence after the split of *Sorghum bicolor* and *S. spontaneum*. These results indicated the gene functional constraint of the orthologous genes.The genes in clade A contained more exons than other clades (Additional file 8). According to phylogeny relationship, Clade A belong to the older group of the *MGT* families. For this reason, the *MGTs* in clade A were speculated to have more events for introns gain/loss according to ‘introns-early’ theory during the lengthy evolutionary process [53, 54].Consequently, we assumed that the evolution of gene structures of MGTs in clade A were mainly derived by the spitted of exon [55].In *Saccharum*, *MTG9* was one of the *MGTs* with highest expression levels in functional photosynthesis region, the maturing zone of leaf (Fig. 5b) and had peak expression in the middle of the night period in *S. spontaneum* but showed no diurnal expression pattern in *S. officinarum.* Moreover, the expression of *SoMGT9* rise sharply in leaf and decrease in stem at ABA treatment. Under GA treatment, *SsMGT9* was induced in leaf, while *SoMGT9* show an unstable change in leaf. *S*uggesting that *MGT9* may involve in the crosstalk of the gene network in response to the two hormones (Additional file 14). *SsMGT9* was predicted to be located in chloroplast (Table 2), and its closest orthologous gene from both Arabidopsis *(ATMGT10, At5G22830*) [56] and rice (*OsMRS2–6, Os03t0684400*) [17] in clade A were found to be located in chloroplast. Moreover, *OsMRS2–6* possessed the Mg^2+^ transport ability to transport Mg into the chloroplast matrix during the day as a chloroplast device [17]. Therefore, we deduced that *MGT9* plays a role as a MGT for chlorophyll formation for photosynthesis and has functional divergence between *S. spontaneum* and *S. officianrum* (Fig. 7).

In clade B, *SsMGT5* was assumed to be retained from σ WGD as *Aco008214.1* distributed the outgroup of subgroup of Poaceae. In *Saccahrum*, *MGT4* and *MGT5* were lowly expressed in the examined tissues and *MGT5* had higher expression levels than *MGT4.* However, in sorghum, *SbMGT4* and *SbMGT5* were highly expressed in the 2 reproductive parts of the pistil and anther, respectively (Additional file 17) [57]. Thus, we can speculate that *MGT*4 and *MGT*5 in sugarcane, as the close relative of sorghum, may not participate in vegetative growth but instead participates in the development of sugarcane reproductive organs. Further experiments would be necessary for testing this hypothesis.

In clade C, *SsMGT1* and *SsMGT2* were assumed to be derived from the ρWGD events based on the reference phylogenetic position of *Aco003216.1*, *SsMGT3* originated from the LCA of angiosperms, *SsMGT10* retained in monocots from the gene duplication event before emergence of angiosperms. Therefore, the evolution history of these 4 *MGT*s could be sorted by age in duplicated descending order: *SsMGT10*, *SsMGT3*, *SsMGT1/ SsMGT2*.*MGT1* was undetectable in all examined tissues of *Sacchaurm*, which is similar to the *SbMGT1* expression pattern in *Sorghum bicolor* (Additional file 17) [57], and *MGT2* had low expression levels in all the detected tissues of *Saccharum*. These results may be caused by gene function redundancy because of the recent ρWGD. *MGT3* had higher expression levels in *S. officnarum* than in *S. spontaneum*, and was abundant in the mature zone of leaf tissues, in mature stems, and probably was induced by light (Figs. 5b and 6). In rice, the closest orthologous genes of *SsMGT3* is *OsMGT1/OsMRS2–2* (*Os01t0869200*) which provides a specific response to Al stress and salt stress in the root [58]. *MGT3* is likely involved in Mg homeostasis in mature stems and in the leaves during the light period in *S. officinarum* but possibly plays a limited role in *S. spontaneum*. *MGT10* was one of the most abundant genes, and its expression was repressed during the light period (Figs. 6 and 7, Additional file 18). These results led us to hypothesize *MGT10* is the predominant *MGT* for maintaining the Mg concentration in chlorophyll during the night period in *Saccharum.* It is interesting that the gene expression correlates with the evolutionary history of the 4 *MGTs* in *Saccharum*, as demonstrated by the results that rank the expression levels as *SsMGT10* > *SsMGT3* > *SsMGT1/SsMGT2.*

In clade D, *SsMGT7* and *SsMGT8* originated from the LCA of the second-round gene duplication in angiosperms by a recent gene duplication event which occurred after the divergence of monocots and dicots. Comparing with other species in the clade, *SsMGT7* and *SsMGT8* have 1 and 2 more exons, and the two gene sizes were larger than those of other *MGTs* in monocotyledon plants. Their structure may be underwent exon/pseudoexon in recent replication events [19], while the intron structure may be generated from the Intron transposition/ transposon insertion/tandem genomic duplication/ Intron transfer or self-splicing type II intron, so we analyzed these two MGTs exon distribution basing on CDS sequence (Additional file 19), the exon 1 in *MGT7* was likely originated from exonization event after the spilt of sorghum and *Saccharum*, similarly, the exon 5 and exon 6 in *MGT8* were indicated to be caused by exonization event after the spilt of sorghum and *Saccharum*. These exonization were predicted to located in the non-conserved regions of the MGTs, thus may not have strong effect on the functional divergence. *MGT7* presented higher expression levels than *MGT8* in the examined tissues of *Saccharum* and have higher expression level in the high sucrose *S. officinarum* than in *S. spontaneum* (Fig. 5)*.* In *Arabidopsis*, the closest orthologous gene, *AtMGT6*(*AT3G58970*), was found to be sensitive to Mg deficiency since the gene was strongly induced by Mg^2+^ deficiency in cortex and epidermal cells, including root hairs [22]. Thus, *MGT7* was assumed to have functional divergence between the 2 *Saccharum* species and may give rise to the response to Mg deficiency in sugarcane.

In clade E, *SsMGT6* was the single gene retained from the common third round of gene duplication event of the *MGT* families (Fig. 4). *MGT6* was the most abundant gene among the *MGT* families and was induced by light with the expression peak at the noon (Fig. 6), suggesting that *MTG6* was the dominant *MGT* to maintain the Mg concentration in chlorophyll during the light period in *Saccharum*. In *Spinacia oleracea. L*., Mg concentration in stroma was observed to be markedly higher in the light period than in the dark period [59]. It is possible that *MGT6* plays a role in the transport of Mg ions to the stroma in the chloroplast matrix during daylight. Of note, the 3 dominant *MGTs, MGT6*, *MGT9* and *MTG10* may have a complementary function for the transport of Mg in the leaf tissue in the light and dark period in *S. spontaneum* (Fig. 7).

## Conclusions

In this research, we identified ten *SsMGTs* in the monoploid genome of *Saccharum spontaneum*. The Ks analysis suggested that *SsMGT* family is an ancient gene family with recent gene duplication events in Poaceae. Based on the phylogenetic analysis, *MGT* genes were predicted to contain at least 5 LCAs before the origin of angiosperms. *SsMGT1/SsMGT2* were observed tooriginate from the recent ρWGD, while *SsMGT7/SsMGT8* were duplicated after the split of dicots and monocots. The truncation of *MGT10* was caused by the pre-mature stop coding. The exon 1 in *MGT7* and the exon 5 and exon 6 in *MGT8* were likely originated from exonization event after the spilt of sorghum and *Saccharum*. These 4 genes were observed to have low expression levels in *Saccharum*, indicating potential functional redundancy caused by the recent duplication of *MGTs*. Three *MGTs, MGT6, MGT9* and *MGT10,* weredominantly expressed among the MGT families in the tissues of *Saccharum. MGT6’s* gene expression was induced in the light period in *S. spontaneum and S. officinarum,* while, *MGT9* and *MTG10* displayed higher expression levels in the dark period in *S. spontaneum*, suggesting that *MGT6* may have a function to complementary *MGT9* and *MTG10* which is regulated by the circadian clock for MGT in the leaf tissues in *S. spontaneum*. The remaining 3 *MGTs*, *MGT3, MGT5* and *MGT7,* had higher expression levels in *S. officinarum* than in *S. spontaneum*, suggesting their functional divergence after the split of *S. spontaneum* and *S. officinarum*. To further understand the function of MGTs in *Saccharum*, experiments such as targeted gene regulation network based on gene expressional profiles, gene over-expression, enzyme activity, and gene knock out technology like CRISPR-Cas9 system, would be necessary. Our study provides the foundation work for the future study of the MGT gene family to characterize the physiological role and molecular mechanisms leading to photosynthesis in sugarcane. The functional complementarity experiment result in*Salmonella typhimurium* strain MM281 suggested that the function of *SsMGTs* issimilar with that of *OSMRS2/MGTs* and *ZmMGTs*, namely transporting magnesium in sugarcane.

## Methods

### Plant materials

Two founding *Saccharum* species, *S. spontaneum* SES208 (Ss, 2n = 8x =64, originated in USA) and *S. officinarum* LAPurple (So, 2n = 8x = 80, originated in USA), were used for the gene expression pattern analysis [60]. The plant material was identified by Irvine JE [61], and These *Saccharum* species were planted in the campus of Fujian Agricultural and Forestry University (Fuzhou, China).

For expression pattern analysis at different developmental stages, samples were collected from 12-month old plants (mature plants) and 9-month old plants (pre-mature plants) for leaf roll, leaf (full expansion leaf), top immature internode (i.e. internode number 3), premature internode (i.e. internode number 9 for ‘LA Purple’ due to short internode, and internode number 6 for SES208 due to long internode) and mature internode (i.e. internode number 15 for ‘LA Purple’ and internode number 9 for SES208 due to long internode – most SES208 plants have about 12 internodes). The internodes were numbered from top to bottom according to the protocol for sugarcane established by Moore [62]. Stem and leaf tissues from seedlings of these 2 species were collected 35 days after planting [63, 64].

Leaves from the mature plants of LAPurple and SES208 were collected to investigate the gene expression under circadian rhythms. In this experiment, from 6 a.m. on March, 22,017 to 4 a.m. on March 3, 2017, samples were collected at 12 time points (6 a.m., 8 a.m., 10 a.m., noon, 2 p.m., 4 p.m., 6 p.m., 8 p.m., 10 p.m., midnight, 2 a.m., 4 a.m.) for RNA isolation. In addition, 7 time points (at 4 h intervals) were collected for a second round of circadian rhythms. The sunset time on 2nd March 2017 was 6 p.m. in Fuzhou. The method was previously described [48, 65].

Similarly, both LAPurple and SES208 were used for the developmental gradient of the leaf experiment. 2 *Saccharum* species plants were grown under the following conditions: light intensity of 350 μmol/m2/sec, 14:10 l/D, 30 °C L/22 °C D and 60% relative humidity. Tissues were collected from 11-day-old second leaves of the *Saccharum* plants, and 15-day-old second leaves of LApurple after planting 3 h into the light period. These leaves were cut into 15 1-cm segments. Samples were pooled from an average of 4 plants per biological replicate and 3 biological replicates in total were prepared. The tissues collection approach followed for this experiment was previously described [65, 66].

The plants for hormone treatment were grown in a growth chamber at 30 °C, 70% RH, and a 14 h:10 h L:D photoperiod. Seedlings were treated with GA (200 μM), ABA (200 μM) for 24, 48, and 96 h. Stem and leaf tissues from the seedlings of the two-sugarcane species were collected from 35-day-old plants [63].

Collected tissues were frozen immediately using liquid nitrogen and stored at − 80 °C prior to RNA isolation. The RNA-seq method that was used in this study are followed by our previous work [63, 64].

### Database search for the MGTs gene family

Ten and 9 MRS2/MGT proteins were obtained from the TAIR [67] and TIGR [68] databases, respectively [16–18]. Putative members of the MRS2/MGT gene family in the sorghum genome were identified using the BLASTP program from the Phytozome V12.1 [69], with *Arabidopsis* and rice MRS2/MGT protein sequences as queries. Sequences with e-values < 10^− 10^ were selected as the preliminary MRS/MGT candidates. Subsequently, we used *SbMGTs* as query sequences to BLAST search the MGT from representative monoploidy genome *S. spontaneum* genome [31].

### Sequence analysis and phylogenetic tree

The CDS sequences of the *MGT* genes were translated into protein sequences by the ExPASy Translate tool [70]. The putatively conserved domains of MGT proteins were detected using BLASTp [71] and InterPro [72]. The isoelectric point and relative molecular mass of the proteins were predicted using ExPASy Compute pI/Mw tool [73]. The exon-intron structures for the MGT genes were drawn using the TBtools [74], the transmembrane domain of the MGTs were predicted by TMHHM [75], and the subcellular location predicted by WOLF PSORT [76].

The amino acid sequences of MRS2/MGT gene family members in 9 monocotyledons (*Zea mays*, *Sorghumbicolor*, *Oryzasativa*, *Brachypodiumdistachyon*, *Ananas comosus, Setaria viridis, Setaria italica, Saccharum cultivar* R570 and *S. spontaneum*), 5 dicotyledons (*Arabidopsisthaliana*, *Carica papaya*, *Amborellatrichopoda*, *Solanumlycopersicum* and *Vitisvinifera*) and 2 Chlorophyta species *(Dunaliella salina* and *Chlamydomonas reinhardtii)* were identified by searching public databases available from various resources.

The evolutionary history was inferred using the Neighbor-Joining(NJ) method [77]. The percentage of replicate trees in which the associated taxa clustered together in the bootstrap test (1000 replicates) are shown next to the branches [78]. The evolutionary distances were computed using the Poisson correction method [79] and were in the units of the number of amino acid substitutions per site. The analysis involved 149 amino acid sequences [80]. The Maximum Likelihood (ML) tree was also constructed by MEGA7, with 100 nonparametric bootstrap replicates. The percentage of trees in which the associated taxa clustered together is shown next to the branches. Initial tree(s) for the heuristic search were obtained automatically by applying Neighbor-Join and BioNJ algorithms to a matrix of pairwise distances estimated using a JTT model, and then the topology with superior log likelihood value was selected.

The non-synonymous (Ka) and synonymous (Ks) substitution ratios of the 10 pairs of orthologous genes from sorghum and sugarcane were calculated by the easy_kaks calculation program [81]. Meanwhile, Fisher’s exact test for small samples was applied to verify the validity of Ka and Ks calculated by this method [82]. The divergence time (T) was calculated by T = Ks/ (2 × 6.1 × 10^− 9^) × 10^− 6^ Mya [83].

### Analysis of the expression profile of MGTs in *Saccharum* based on RNA-seq

Five μg total RNA of each sample was used for the construction of cDNA libraries. The cDNA libraries were prepared using Illumina® TruSeq™ RNA Sample Preparation Kit (RS-122–2001(2), Illumina) according to the manufacturer’s protocol. The RNA-seq libraries were pooled and sequenced with 100 single reads on Illumina HiSeq2500 at the Center for Genomics and Biotechnology at the Fujian Agriculture and Forestry University.

Raw data was aligned to reference gene models using TRINITY [84]. Trinity combines 3 independent software modules: Inchworm, Chrysalis, and Butterfly, applied sequentially to process large volumes of RNA-seq reads. Trinity partitions the sequence data into many individual de Bruijn graphs, each representing the transcriptional complexity at a given gene or locus, and then processes each graph independently to extract full-length splicing isoforms and to tease apart transcripts derived from paralogous genes. There is 59.84% overall alignment rate in this study.

### Sub-cellular localization analysis

GATEWAY technology (Invitrogen) was used in this study, a translational fusion between each isolated gene and GFP was designed. The attB recombination sites: 5′- GGGGACAAGTTTGTACAAAAAAGCAGGCTTG (for the forward primer) and 5′- GGGGACCACTTTGTACAAGAAAGCTGGGTG (for the reverse primer), PCR products were used in a BP clonase reaction for recombination into the p207-DONOR vector. Positive clones were then used in an LR clonase reaction for recombination into the pMDC84 destination vector, which contains two copies of the 35S promoter [85]. *A. tumefaciens* was transformed with the expression vectors and then agroinfiltrated in *Nicotiana tabacum* cv. Petite Havana (SR1) leaves. Middle leavesfrom seven-week-old plants were infiltrated with an *A. tumefaciens* culture grown for 2 days at 28 °C, the leaf was used to localize GFP fluorescence by a confocal microscope (Leica TCS SP8X DLS).

### Validation of RPKM values for MGT genes using RT-qPCR

RNA (≤1 μg) from each sample was reverse-transcribed to cDNA using the Reverse Transcriptase Kit (Takara, Code, RR047A) in a 20 ml reaction volume with 1 ml of random primers and 1 ml of mixed poly-dT primers (18–23 nt). The cDNA was diluted 1:19 in water for further RT-qPCR experiments. Specific primers (S1) were designed by Integrated DNA technologies [86]. The TransStart Tip Green RT-qPCR SuperMix kit was used for RT-qPCR, and the reaction cycle was: 95 °C for 30s, 40 cycles of 95 °C for 5 s, and 60 °C for 30s, and 95°C for 10s. The consistency of the melting curve demonstrated the reliability of RT-qPCR results. In order to normalize the expression levels, 2 constitutively expressed genes, glyceraldehyde-3-phosphate dehydrogenase gene (GAPDH) and the eukaryotic elongation factor 1a (eEF-1a) were used as reference genes, each sample had 3 biological replicates and 4 technical replicates. The relative expression levels for each *MGT* gene in different tissues of 2 sugarcane species were calculated using the 2^-ΔΔCt^ method [87].

## Additional files

Additional file 1: The BLASTP result of ShMGTs by using SsMGTs and SbMGTs as query. (XLS 112 kb)

Additional file 2: Divergence between a pair of tandem duplicated genes (*Sh06_t004330* and *Sh06_t004340*) and the identity between these two genes. (PPTX 78 kb)

Additional file 3: Transmembrane Protein Topology prediction of SsMGTs with a Hidden Markov Model. (DOC 71 kb)

Additional file 4: The similarity between SsMGT proteins was calculated by NCBI BLASTP. (DOC 34 kb)

Additional file 5: Amino acid sequences of 16 species. (FASTA 133 kb)

Additional file 6: Phylogenetic analysis of MGTs from 16 plant species by Maximum Likelihood method. (PDF 53 kb)

Additional file 7: A schematic diagram for the relationship of the 5 clades of the phylogenetic tree constructed by NJ method. (DOC 170 kb)

Additional file 8: The exon number of *MGTs*. (XLS 38 kb)

Additional file 9: The proportion of the same number of exons in all *MGTs*. (DOC 25 kb)

Additional file 10: The non-synonymous (Ka) and synonymous (Ks) substitution ratios of each *MGTs* from 10 representative plants. (XLSX 804 kb)

Additional file 11: RT-qPCR of *SsMGT*. (DOC 51 kb)

Additional file 12 : qRT-PCR primers. (DOC 29 kb)

Additional file 13: The expression levels of MGTs in *S. officinarum* based on RPKM in parenchyma cells and sclerenchyma cells from *S. officinarum*. (DOC 32 kb)

Additional file 14: The expression patterns of *MGTs* with hormone treatment. (PDF 1945 kb)

Additional file 15: Expression patterns of *MGT* genes across leaf gradient segments in maize and rice [88]. (XLSX 24 kb)

Additional file 16: Complementation of the MM281 mutant by *SsMGT3*. (PDF 418 kb)

Additional file 17: The expression patterns of MGTs in *Sorghumbicolor* based on qTeller [57, 89]. Abbreviation: Lvs: Young leaves were harvested 20 days after sowing; stg1inf: Primodial inflorescences were harvested 10 days before flower emergence; stg2inf: Whole inflorescences were harvested at the time of flower emergence; anth: Whole anthers were harvested at the time anthesis; pist: Pistils were harvested at the time anthesis; sd5: Whole seeds were harvested 5 days after pollination; sd10: Whole seeds were harvested 10 days after pollination; emb: Developing embryos were harvested 25 days after pollination; endo: Developing endosperms were harvested 25 days after pollination. (DOC 58 kb)

Additional file 18: Subcellular localization of *SoMGT10*, *SoMGT10* were localized to the chloroplast in tobacco. *35S:SoMGT10–GFP* constructs were introduced into tobacco, and fluorescence was visualized by confocal laser microscopy. Bars = 25 μm. (PPTX 7792 kb)

Additional file 19: The exon-intron structures of *MGT* genes in sugarcane, sorghum, maize, millet, green bristle grass and rice. The corresponding exons with similar sequences based on sequence alignments were indicated with solid lines. Sequence similarity values are shown as percentage. Exons that had experienced exon gain events are labeled with blue box, and exonization/ pseudoexonization events are labeled with red box. For gene IDs, Ss indicates *S. spontaneum*, Sh indicates *Saccharum* hybrids, Sb indicates *Sorghum bicolor*, GRMZM indicates *Zea mays*, Seita indicates *Setaria italic*, Sevir indicates *Setaria viridis* and Os indicates *Oryza sativa*. A. *SsMGT7*, B. *SsMGT8*. (PDF 13824 kb)

## Abbreviations

eEF-1a: Eukaryotic elongation factor 1a
GAPDH: Glyceraldehyde-3-phosphate dehydrogenase gene
GFP: Green fluorescent protein
Ka: Non-synonymous
Ks: Synonymous
LCA: Last common ancestor
MGT: Magnesium transport
ORFs: Open reading frames
PCR: Polymerase chain reaction
RNA-seq: RNA Sequencing
RPKM: Reads Per Kilobase per Million mapped reads
RT-qPCR: Reverse transcription quantitative PCR
WGD: Whole genome duplication
