# Supplementary figures and images for: Comparative genomics revealed the gene evolution and functional divergence of magnesium transporter families in Saccharum
Source: BMC Genomics. 2019 Jan 24;20:83. doi: 10.1186/s12864-019-5437-3 (PMC6345045; doi:10.1186/s12864-019-5437-3)

## Slide 1
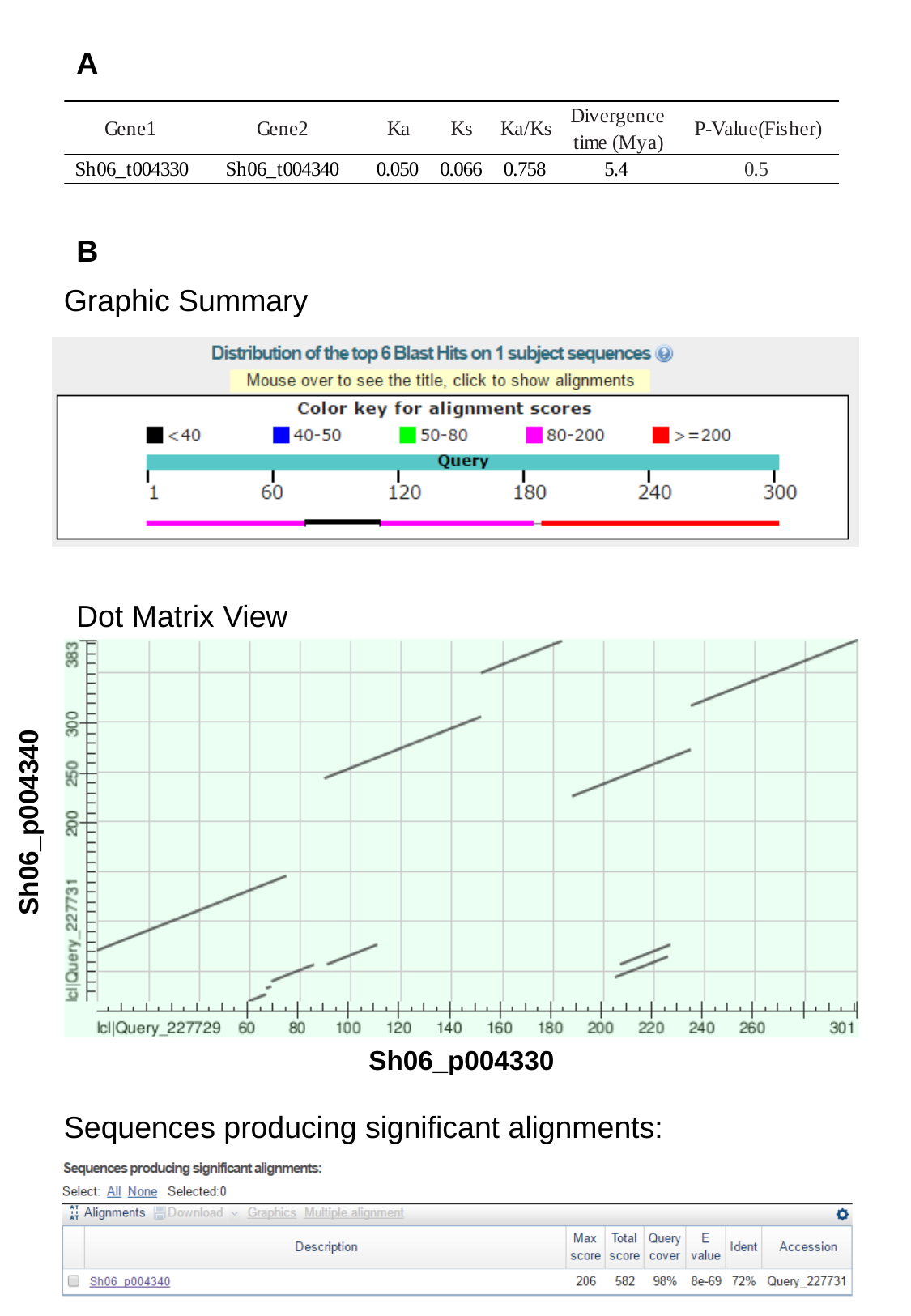

A
B
Graphic Summary
Dot Matrix View
Sh06_p004340
Sh06_p004330
Sequences producing significant alignments:

Supplement: Supplementary file 2 — Divergence between a pair of tandem duplicated genes (Sh06_t004330 and Sh06_t004340) and the identity between these two genes. (PPTX 78 kb) [file 12864_2019_5437_MOESM2_ESM.pptx]

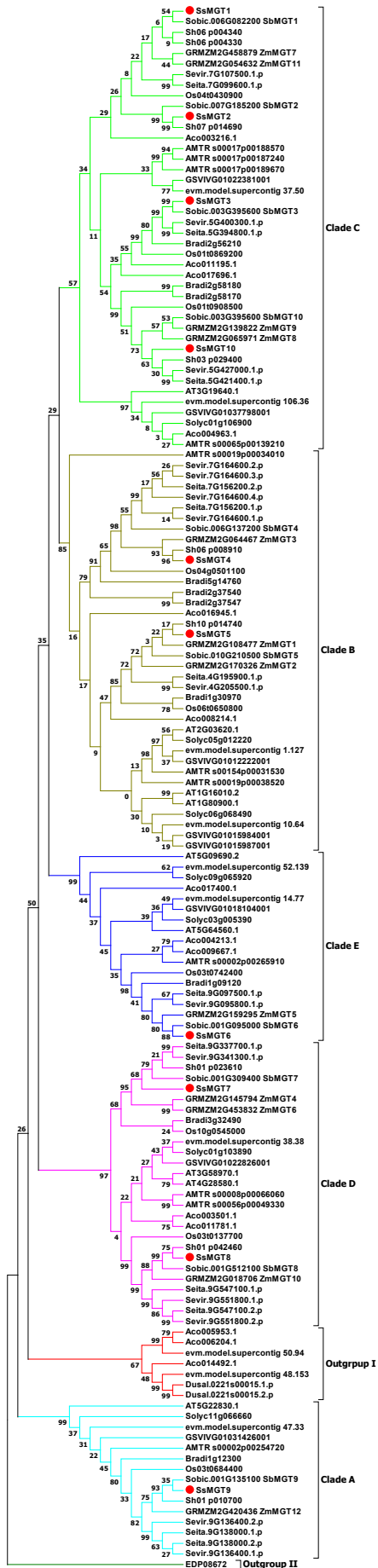

Supplement: Supplementary file 6 — Phylogenetic analysis of MGTs from 16 plant species by Maximum Likelihood method. (PDF 53 kb) [file 12864_2019_5437_MOESM6_ESM.pdf]

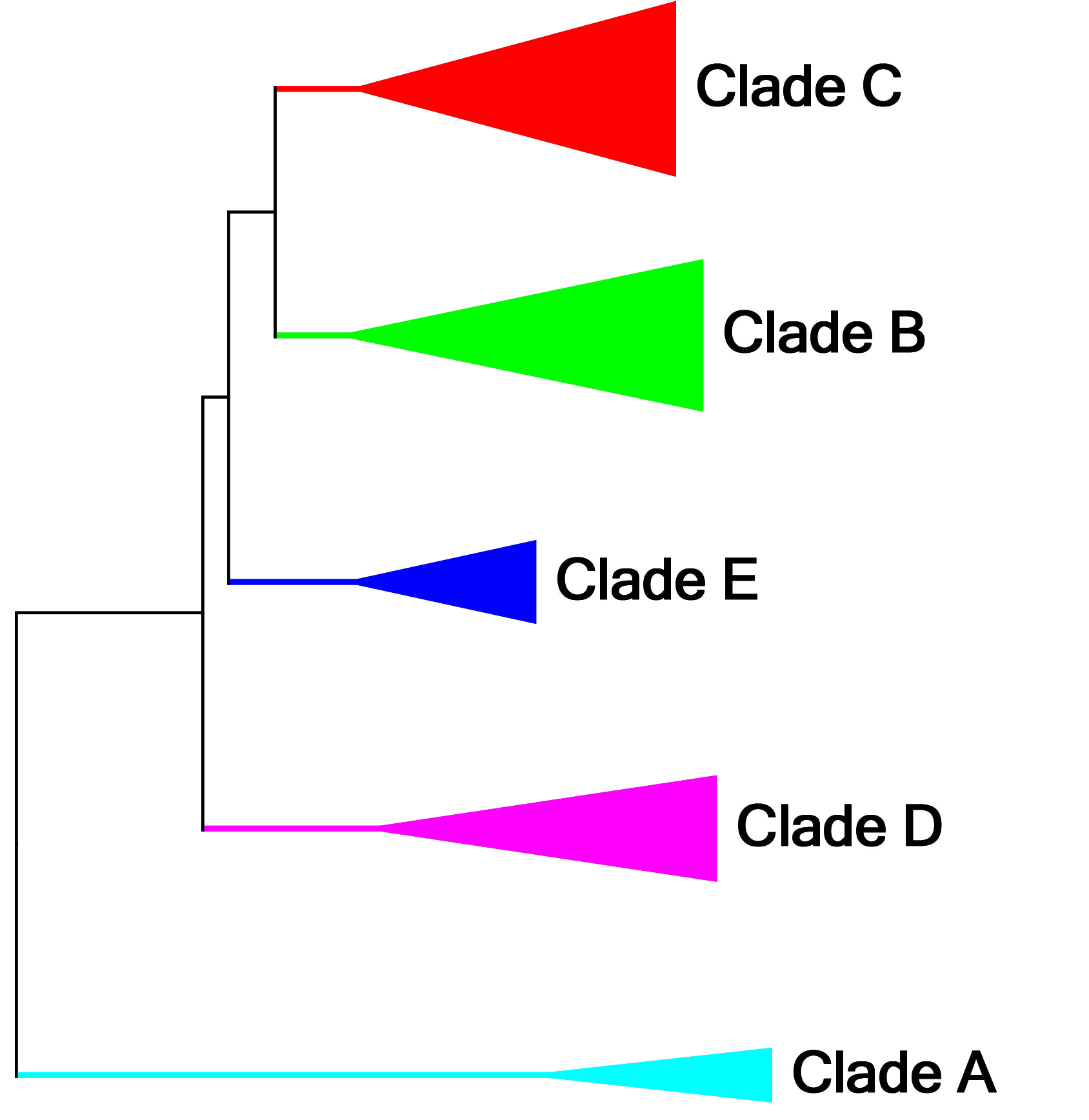

Supplement: Supplementary file 7 — A schematic diagram for the relationship of the 5 clades of the phylogenetic tree constructed by NJ method. (DOC 170 kb) [file 12864_2019_5437_MOESM7_ESM.doc]

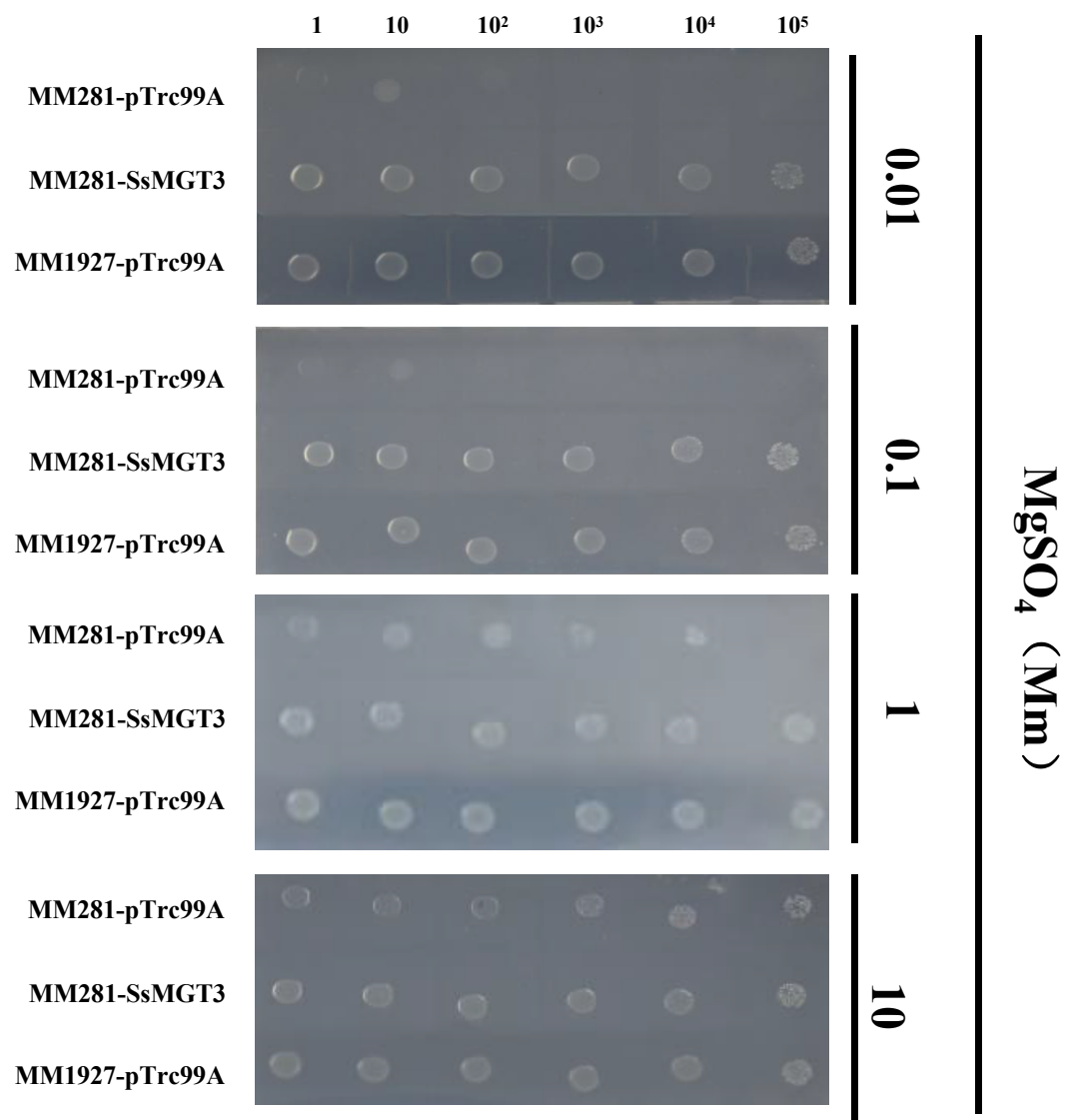

Supplement: Supplementary file 16 — Complementation of the MM281 mutant by SsMGT3. (PDF 418 kb) [file 12864_2019_5437_MOESM16_ESM.pdf]

## Slide 1
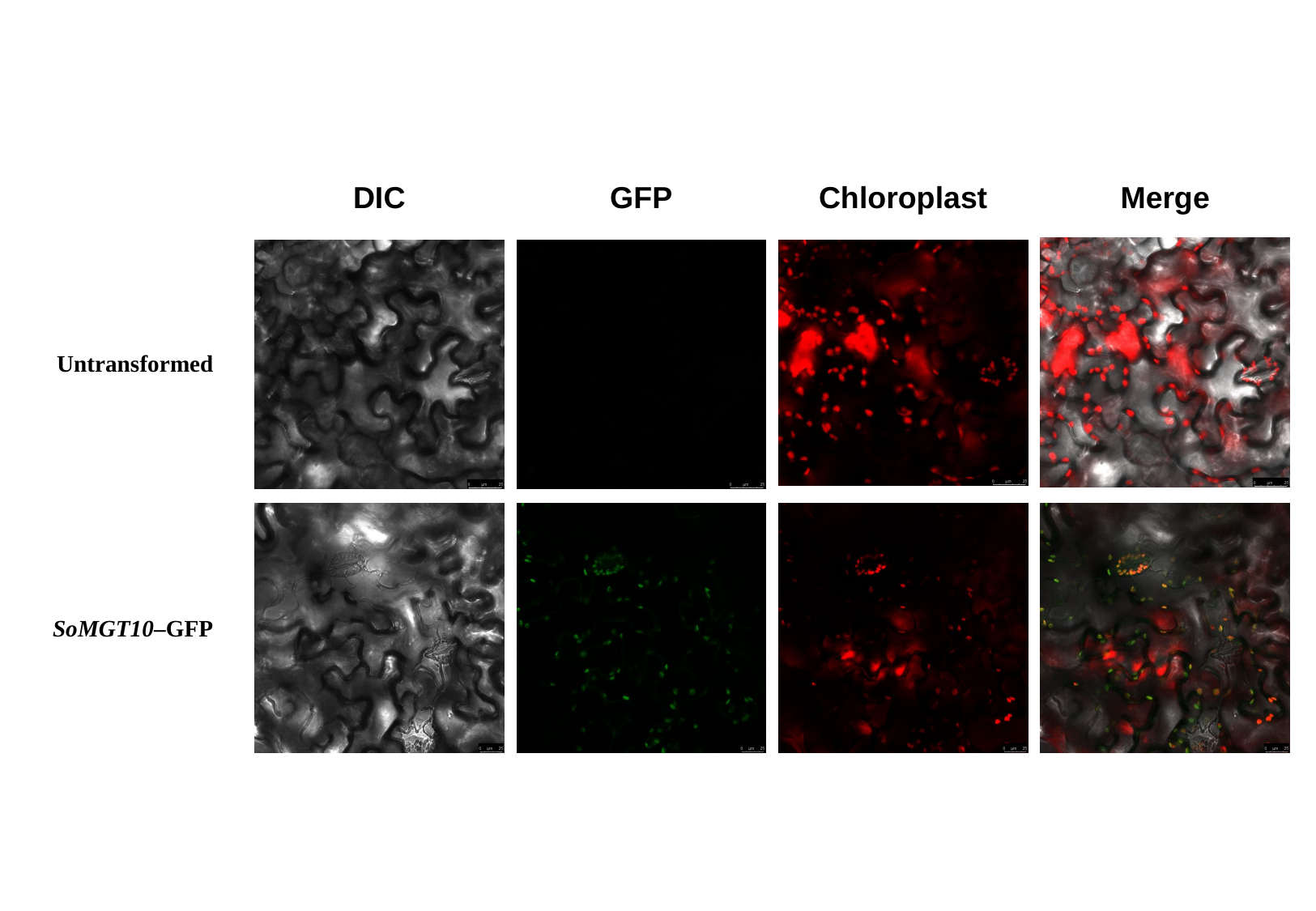

DIC
GFP
Chloroplast
Merge
Untransformed
SoMGT10–GFP

Supplement: Supplementary file 18 — Subcellular localization of SoMGT10, SoMGT10 were localized to the chloroplast in tobacco. 35S:SoMGT10–GFP constructs were introduced into tobacco, and fluorescence was visualized by confocal laser microscopy. Bars = 25 μm. (PPTX 7792 kb) [file 12864_2019_5437_MOESM18_ESM.pptx]

**A**

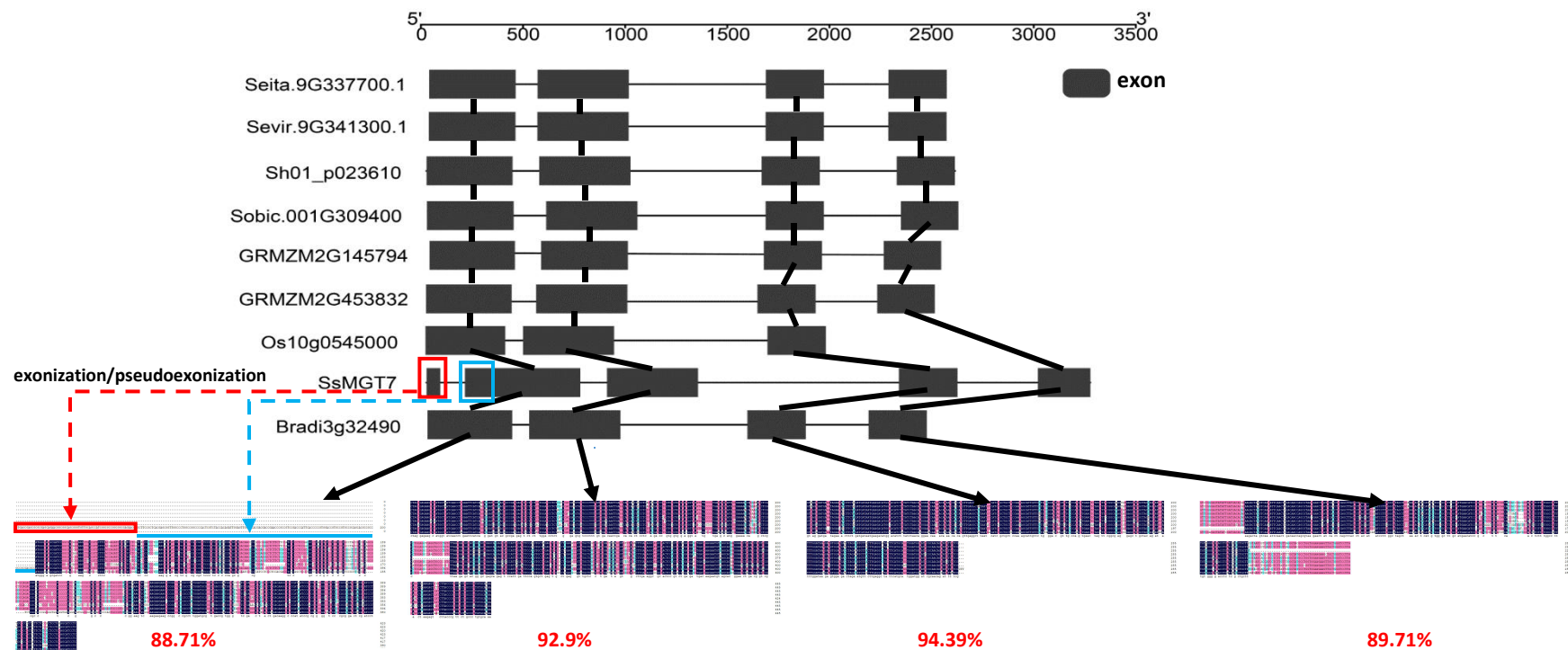

**B**

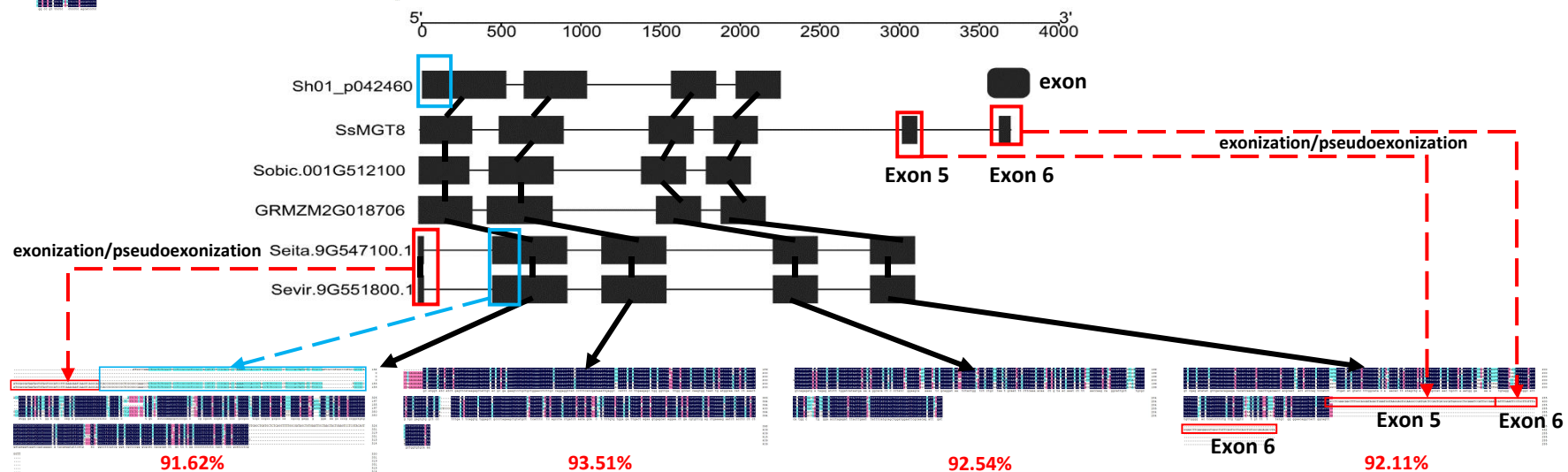

Supplement: Supplementary file 19 — The exon-intron structures of MGT genes in sugarcane, sorghum, maize, millet, green bristle grass and rice. The corresponding exons with similar sequences based on sequence alignments were indicated with solid lines. Sequence similarity values are shown as percentage. Exons that had experienced exon gain events are labeled with blue box, and exonization/ pseudoexonization events are labeled with red box. For gene IDs, Ss indicates S. spontaneum, Sh indicates Saccharum hybrids, Sb indicates Sorghum bicolor, GRMZM indicates Zea mays, Seita indicates Setaria italic, Sevir indicates Setaria viridis and Os indicates Oryza sativa. A. SsMGT7, B. SsMGT8. (PDF 13824 kb) [file 12864_2019_5437_MOESM19_ESM.pdf]
