# Supplementary material for: Comparative genomics revealed the gene evolution and functional divergence of magnesium transporter families in Saccharum
Source: BMC Genomics. 2019 Jan 24;20:83. doi: 10.1186/s12864-019-5437-3 (PMC6345045; doi:10.1186/s12864-019-5437-3)

# SsMGT 1 Length: 400

# SsMGT 1 Number of predicted TMHs: 1

# SsMGT 1 Exp number of AAs in TMHs: 22.37814

# SsMGT 1 Exp number, first 60 AAs: 0.08387

# SsMGT 1 Total prob of N-in: 0.57617

SsMGT 1 TMHMM2.0 inside 1 344

SsMGT 1 TMHMM2.0 TMhelix 345 367

SsMGT 1 TMHMM2.0 outside 368 400


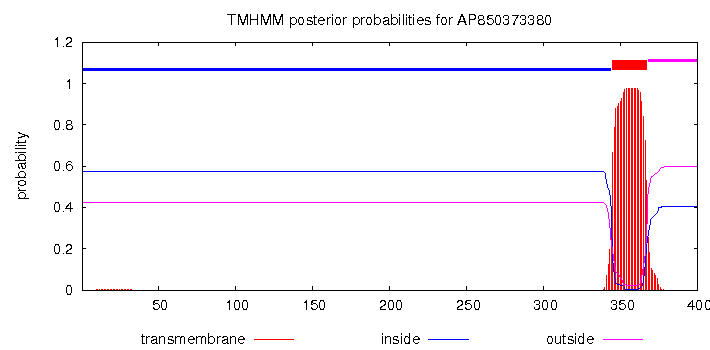


# SsMGT 2 Length: 410

# SsMGT 2 Number of predicted TMHs: 2

# SsMGT 2 Exp number of AAs in TMHs: 46.3513

# SsMGT 2 Exp number, first 60 AAs: 0.00863

# SsMGT 2 Total prob of N-in: 0.79146

SsMGT 2 TMHMM2.0 inside 1 343

SsMGT 2 TMHMM2.0 TMhelix 344 366

SsMGT 2 TMHMM2.0 outside 367 380

SsMGT 2 TMHMM2.0 TMhelix 381 403

SsMGT 2 TMHMM2.0 inside 404 410


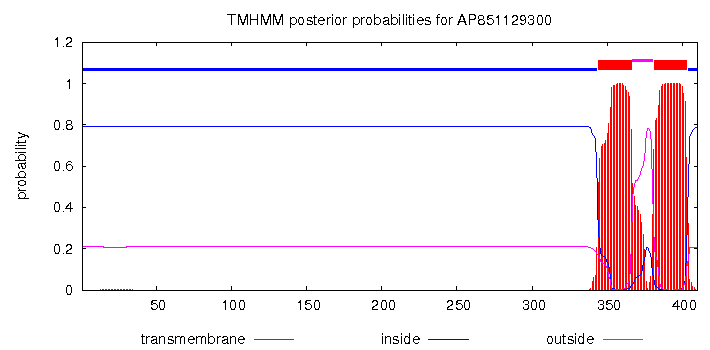


# SsMGT 3 Length: 410

# SsMGT 3 Number of predicted TMHs: 2

# SsMGT 3 Exp number of AAs in TMHs: 45.31452

# SsMGT 3 Exp number, first 60 AAs: 0.0011

# SsMGT 3 Total prob of N-in: 0.02189

SsMGT 3 TMHMM2.0 outside 1 335

SsMGT 3 TMHMM2.0 TMhelix 336 358

SsMGT 3 TMHMM2.0 inside 359 378

SsMGT 3 TMHMM2.0 TMhelix 379 401

SsMGT 3 TMHMM2.0 outside 402 410


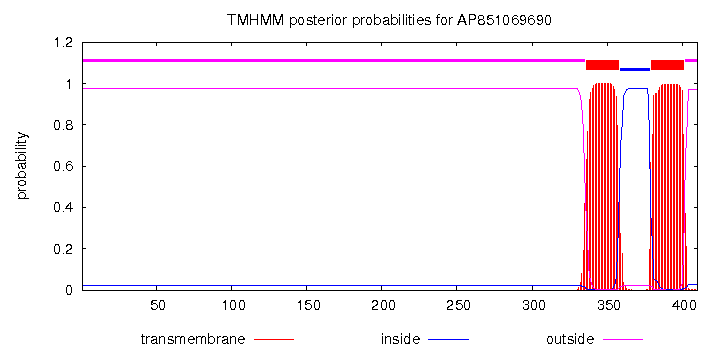


# SsMGT 4 Length: 399

# SsMGT 4 Number of predicted TMHs: 1

# SsMGT 4 Exp number of AAs in TMHs: 22.12837

# SsMGT 4 Exp number, first 60 AAs: 0.00047

# SsMGT 4 Total prob of N-in: 0.00483

SsMGT 4 TMHMM2.0 outside 1 368

SsMGT 4 TMHMM2.0 TMhelix 369 391

SsMGT 4 TMHMM2.0 inside 392 399


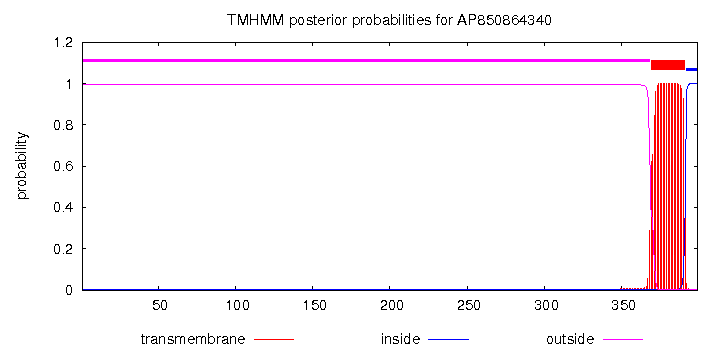


# SsMGT 5 Length: 446

# SsMGT 5 Number of predicted TMHs: 2

# SsMGT 5 Exp number of AAs in TMHs: 44.8323

# SsMGT 5 Exp number, first 60 AAs: 0

# SsMGT 5 Total prob of N-in: 0.37735

SsMGT 5 TMHMM2.0 inside 1 381

SsMGT 5 TMHMM2.0 TMhelix 382 401

SsMGT 5 TMHMM2.0 outside 402 415

SsMGT 5 TMHMM2.0 TMhelix 416 438

SsMGT 5 TMHMM2.0 inside 439 446


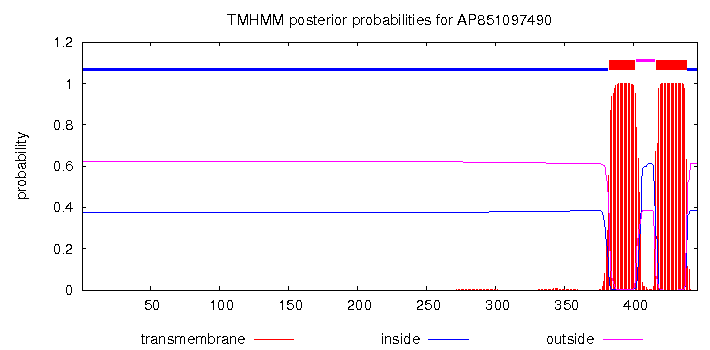


# SsMGT 6 Length: 357

# SsMGT 6 Number of predicted TMHs: 2

# SsMGT 6 Exp number of AAs in TMHs: 44.63787

# SsMGT 6 Exp number, first 60 AAs: 0.00024

# SsMGT 6 Total prob of N-in: 0.51272

SsMGT 6 TMHMM2.0 inside 1 292

SsMGT 6 TMHMM2.0 TMhelix 293 315

SsMGT 6 TMHMM2.0 outside 316 324

SsMGT 6 TMHMM2.0 TMhelix 325 347

SsMGT 6 TMHMM2.0 inside 348 357


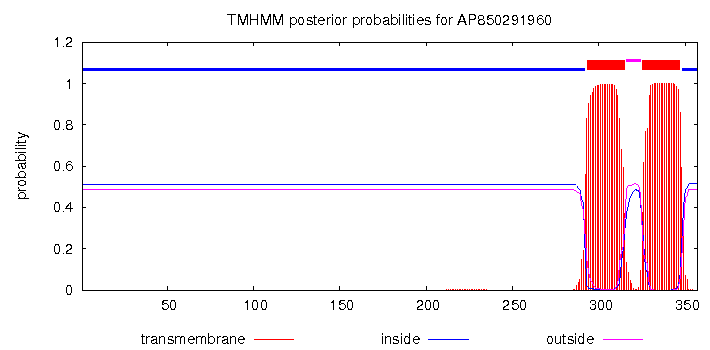


# SsMGT 7 Length: 539

# SsMGT 7 Number of predicted TMHs: 2

# SsMGT 7 Exp number of AAs in TMHs: 45.60494

# SsMGT 7 Exp number, first 60 AAs: 0.47082

# SsMGT 7 Total prob of N-in: 0.03579

SsMGT 7 TMHMM2.0 outside 1 474

SsMGT 7 TMHMM2.0 TMhelix 475 497

SsMGT 7 TMHMM2.0 inside 498 509

SsMGT 7 TMHMM2.0 TMhelix 510 532

SsMGT 7 TMHMM2.0 outside 533 539


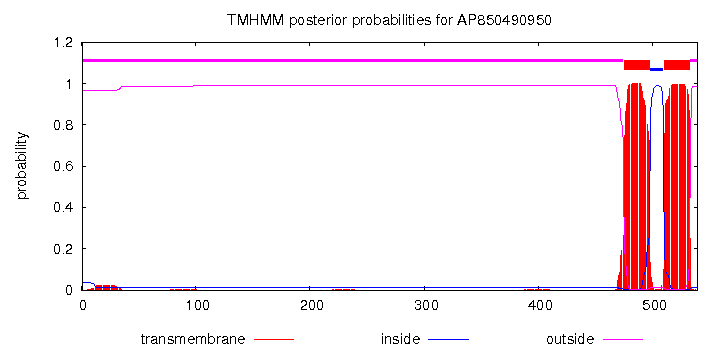


# SsMGT 8 Length: 492

# SsMGT 8 Number of predicted TMHs: 2

# SsMGT 8 Exp number of AAs in TMHs: 45.91995

# SsMGT 8 Exp number, first 60 AAs: 0.00342

# SsMGT 8 Total prob of N-in: 0.51168

SsMGT 8 TMHMM2.0 inside 1 368

SsMGT 8 TMHMM2.0 TMhelix 369 391

SsMGT 8 TMHMM2.0 outside 392 405

SsMGT 8 TMHMM2.0 TMhelix 406 428

SsMGT 8 TMHMM2.0 inside 429 492


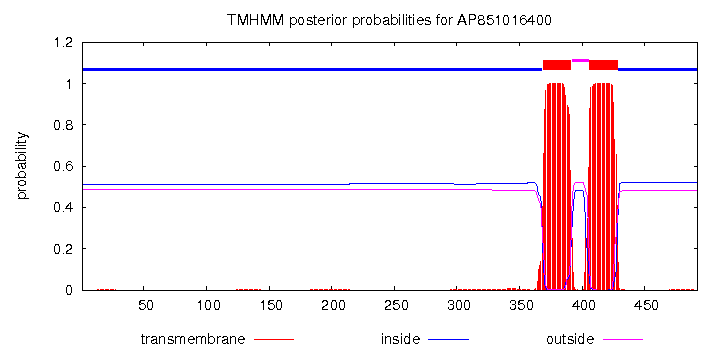


# SsMGT 9 Length: 428

# SsMGT 9 Number of predicted TMHs: 2

# SsMGT 9 Exp number of AAs in TMHs: 47.95884

# SsMGT 9 Exp number, first 60 AAs: 0.01084

# SsMGT 9 Total prob of N-in: 0.29186

SsMGT 9 TMHMM2.0 outside 1 365

SsMGT 9 TMHMM2.0 TMhelix 366 385

SsMGT 9 TMHMM2.0 inside 386 396

SsMGT 9 TMHMM2.0 TMhelix 397 419

SsMGT 9 TMHMM2.0 outside 420 428


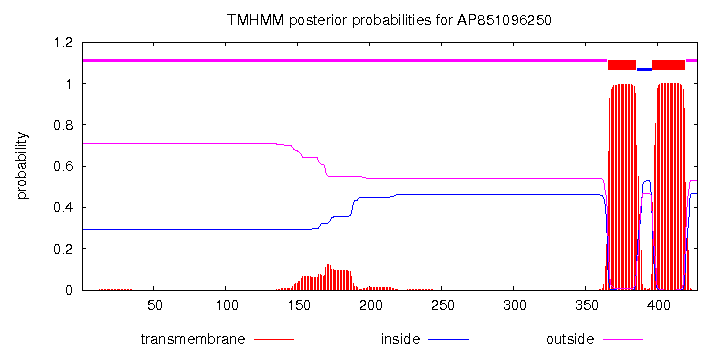


# SsMGT 10 Length: 290

# SsMGT 10 Number of predicted TMHs: 0

# SsMGT 10 Exp number of AAs in TMHs: 0.00035

# SsMGT 10 Exp number, first 60 AAs: 0.00018

# SsMGT 10 Total prob of N-in: 0.01395

SsMGT 10 TMHMM2.0 outside 1 290


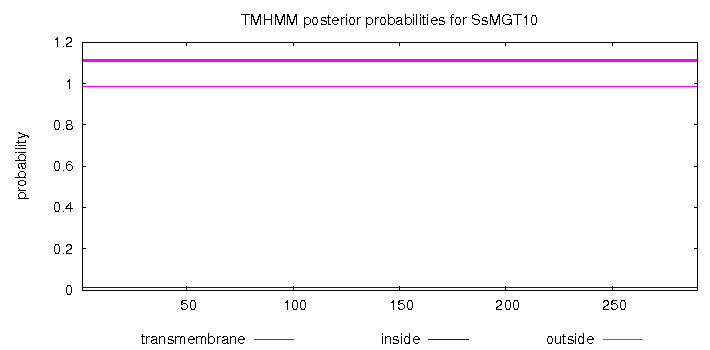

Supplement: Supplementary file 3 — Transmembrane Protein Topology prediction of SsMGTs with a Hidden Markov Model. (DOC 71 kb) [file 12864_2019_5437_MOESM3_ESM.doc]
