# Supplementary material for: Comparative genomics revealed the gene evolution and functional divergence of magnesium transporter families in Saccharum
Source: BMC Genomics. 2019 Jan 24;20:83. doi: 10.1186/s12864-019-5437-3 (PMC6345045; doi:10.1186/s12864-019-5437-3)
Supplement: Supplementary file 4 — The similarity between SsMGT proteins was calculated by NCBI BLASTP. (DOC 34 kb) [file 12864_2019_5437_MOESM4_ESM.doc]

|  | SsMGT1 | SsMGT2 | SsMGT3 | SsMGT4 | SsMGT5 | SsMGT6 | SsMGT7 | SsMGT8 | SsMGT9 | SsMGT10 |
| --- | --- | --- | --- | --- | --- | --- | --- | --- | --- | --- |
| SsMGT2 | 63% | - | - | - | - | - | - | - | - | - |
| SsMGT3 | 50% | 46% | - | - | - | - | - | - | - | - |
| SsMGT4 | 40% | 36% | 40% | - | - | - | - | - | - | - |
| SsMGT5 | 45% | 43% | 48% | 55% | - | - | - | - | - | - |
| SsMGT6 | 41% | 38% | 40% | 37% | 40% | - | - | - | - | - |
| SsMGT7 | 37% | 32% | 37% | 34% | 41% | 39% | - | - | - | - |
| SsMGT8 | 35% | 33% | 32% | 35% | 40% | 36% | 56% | - | - | - |
| SsMGT9 | 23% | 23% | 22% | 21% | 38% | 24% | 22% | 21% | - | - |
| SsMGT10 | 51% | 48% | 47% | 57% | 59% | 40% | 36% | 32% | 31% | - |

**Additional File 4 The similarity between MGT proteins in *Saccharum* was calculated by NCBI BLASTP**
