# Supplementary material for: Comparative genomics revealed the gene evolution and functional divergence of magnesium transporter families in Saccharum
Source: BMC Genomics. 2019 Jan 24;20:83. doi: 10.1186/s12864-019-5437-3 (PMC6345045; doi:10.1186/s12864-019-5437-3)
Supplement: Supplementary file 9 — The proportion of the same number of exons in all MGTs. (DOC 25 kb) [file 12864_2019_5437_MOESM9_ESM.doc]

**Additional File 9:** The proportion of the same number of exons in all *MGTs*
