# Supplementary material for: Comparative genomics revealed the gene evolution and functional divergence of magnesium transporter families in Saccharum
Source: BMC Genomics. 2019 Jan 24;20:83. doi: 10.1186/s12864-019-5437-3 (PMC6345045; doi:10.1186/s12864-019-5437-3)
Supplement: Supplementary file 12 — : qRT-PCR primers. (DOC 29 kb) [file 12864_2019_5437_MOESM12_ESM.doc]

| Gene | primer |
| --- | --- |
| SsMGT6F(qPCR) | GACGACATGGCTGATCTTTACT |
| SsMGT6R(qPCR) | GGCTCTGCTTGCTCTTGATA |
| SsMGT9F(qPCR) | TGGTACGTGAGCAAGCTATTT |
| SsMGT9R(qPCR) | CTTTCCTCCTGGGCTGTTATAG |
| SsMGT10F(qPCR) | TTGGATGAGCTGACATCAAAGA |
| SsMGT10R(qPCR) | ACCTTCTGTACTCGACCAGATA |

**Additional File 12:** qRT-PCR primers.
