# Supplementary material for: Comparative genomics revealed the gene evolution and functional divergence of magnesium transporter families in Saccharum
Source: BMC Genomics. 2019 Jan 24;20:83. doi: 10.1186/s12864-019-5437-3 (PMC6345045; doi:10.1186/s12864-019-5437-3)
Supplement: Supplementary file 13 — The expression levels of MGTs in S. officinarum based on RPKM in parenchyma cells and sclerenchyma cells from S. officinarum. (DOC 32 kb) [file 12864_2019_5437_MOESM13_ESM.doc]

| Gene | P | S |
| --- | --- | --- |
| SoMGT1 | 0.00 | 0.00 |
| SoMGT2 | 0.94 | 4.49 |
| SoMGT3 | 30.02 | 27.72 |
| SoMGT4 | 3.98 | 0.80 |
| SoMGT5 | 12.00 | 9.93 |
| SoMGT6 | 71.04 | 84.61 |
| SoMGT7 | 26.80 | 23.43 |
| SoMGT8 | 3.03 | 3.16 |
| SoMGT9 | 10.62 | 2.30 |
| SoMGT10 | 107.25 | 93.38 |
