# Supplementary material for: Comparative genomics revealed the gene evolution and functional divergence of magnesium transporter families in Saccharum
Source: BMC Genomics. 2019 Jan 24;20:83. doi: 10.1186/s12864-019-5437-3 (PMC6345045; doi:10.1186/s12864-019-5437-3)
Supplement: Supplementary file 14 — The expression patterns of MGTs with hormone treatment. (PDF 1945 kb) [file 12864_2019_5437_MOESM14_ESM.pdf]

A

Gene / samples	MGT1				MGT2				MGT3				MGT4				MGT5			
	Ss	Change fold	So	Change fold	Ss	Change fold	So	Change fold	Ss	Change fold	So	Change fold	Ss	Change fold	So	Change fold	Ss	Change fold	So	Change fold
Leaf-C-24h	0.00	NA	0.00	NA	1.52	1.00	1.63	1.00	12.10	1.00	13.58	1.00	10.50	1.00	11.98	1.00	26.21	1.00	27.46	1.00
Leaf-C-48h	0.00	NA	0.00	NA	1.52	1.00	1.01	1.00	7.01	1.00	15.93	1.00	10.16	1.00	4.80	1.00	28.41	1.00	15.52	1.00
Leaf-C-96h	0.00	NA	0.00	NA	2.25	1.00	0.91	1.00	9.89	1.00	12.45	1.00	9.64	1.00	6.87	1.00	27.43	1.00	24.88	1.00
Stem-C-24h	0.00	NA	0.00	NA	5.67	1.00	3.74	1.00	4.01	1.00	10.05	1.00	6.05	1.00	2.60	1.00	16.98	1.00	14.12	1.00
Stem-C-48h	0.00	NA	0.00	NA	2.82	1.00	1.54	1.00	11.90	1.00	8.63	1.00	6.45	1.00	1.94	1.00	16.04	1.00	10.14	1.00
Stem-C-96h	0.00	NA	0.00	NA	4.58	1.00	1.44	1.00	13.84	1.00	13.52	1.00	4.04	1.00	2.90	1.00	10.18	1.00	9.66	1.00
Leaf-ABA-24h	0.00	NA	0.00	NA	6.95	4.57	5.03	3.09	6.22	0.51	7.79	0.57	6.47	0.62	7.59	0.63	16.65	0.64	15.05	0.55
Leaf-ABA-48h	0.00	NA	0.00	NA	3.59	2.36	0.83	0.82	4.75	0.68	50.53	3.17	3.61	0.36	18.82	3.92	31.07	1.09	13.47	0.87
Leaf-ABA-96h	0.00	NA	0.00	NA	0.80	0.36	2.12	2.33	10.44	1.06	52.06	4.18	2.97	0.31	10.05	1.46	19.26	0.70	16.71	0.67
Stem-ABA-24h	0.00	NA	0.00	NA	2.24	0.40	1.39	0.37	10.34	2.58	15.39	1.53	9.52	1.57	8.59	3.30	12.88	0.76	12.81	0.91
Stem-ABA-48h	0.85	NA	0.00	NA	1.77	0.63	0.80	0.52	9.35	0.79	23.37	2.71	6.18	0.96	10.92	5.63	15.74	0.98	13.73	1.35
Stem-ABA-96h	0.00	NA	0.00	NA	4.63	1.01	1.83	1.27	9.41	0.68	22.90	1.69	6.86	1.70	10.99	3.79	15.52	1.52	16.28	1.69
Leaf-GA-24h	0.00	NA	0.00	NA	2.14	1.41	10.25	6.29	9.59	0.79	13.45	0.99	8.96	0.85	4.67	0.39	11.15	0.43	16.70	0.61
Leaf-GA-48h	0.00	NA	0.00	NA	2.63	1.73	4.31	4.27	7.57	1.08	25.26	1.59	1.53	0.15	16.27	3.39	26.46	0.93	23.90	1.54
Leaf-GA-96h	0.00	NA	0.00	NA	2.00	0.89	1.66	1.82	11.00	1.11	30.72	2.47	2.96	0.31	23.51	3.42	23.63	0.86	26.23	1.05
Stem-GA-24h	0.00	NA	0.00	NA	1.38	0.24	4.15	1.11	7.99	1.99	11.67	1.16	8.33	1.38	8.08	3.11	12.91	0.76	19.16	1.36
Stem-GA-48h	0.00	NA	0.00	NA	1.45	0.51	3.56	2.31	5.72	0.48	15.74	1.82	5.66	0.88	10.31	5.31	13.98	0.87	25.03	2.47
Stem-GA-96h	0.00	NA	0.00	NA	8.36	1.83	2.98	2.07	11.81	0.85	9.29	0.69	10.17	2.52	10.49	3.62	17.98	1.77	26.21	2.71

Gene / samples	MGT6				MGT7				MGT8				MGT9				MGT10			
	Ss	Change fold	So	Change fold	Ss	Change fold	So	Change fold	Ss	Change fold	So	Change fold	Ss	Change fold	So	Change fold	Ss	Change fold	So	Change fold
Leaf-C-24h	90.13	1.00	71.91	1.00	21.83	1.00	21.94	1.00	1.80	1.00	2.96	1.00	29.21	1.00	38.34	1.00	43.33	1.00	81.50	1.00
Leaf-C-48h	71.70	1.00	68.96	1.00	18.98	1.00	28.56	1.00	2.19	1.00	3.37	1.00	22.18	1.00	23.33	1.00	56.93	1.00	71.21	1.00
Leaf-C-96h	79.12	1.00	74.57	1.00	16.32	1.00	24.45	1.00	3.86	1.00	4.38	1.00	61.68	1.00	43.05	1.00	42.03	1.00	66.12	1.00
Stem-C-24h	50.13	1.00	57.15	1.00	19.91	1.00	19.26	1.00	5.68	1.00	4.82	1.00	23.29	1.00	44.47	1.00	58.10	1.00	121.90	1.00
Stem-C-48h	59.77	1.00	47.13	1.00	23.77	1.00	23.51	1.00	5.28	1.00	11.04	1.00	21.13	1.00	36.41	1.00	77.31	1.00	160.22	1.00
Stem-C-96h	48.18	1.00	32.78	1.00	30.26	1.00	20.46	1.00	4.51	1.00	6.97	1.00	24.32	1.00	33.39	1.00	67.46	1.00	146.30	1.00
Leaf-ABA-24h	72.28	0.80	69.70	0.97	11.50	0.53	29.79	1.36	7.55	4.19	1.45	0.49	55.60	1.90	15.68	0.41	43.61	1.01	70.38	0.86
Leaf-ABA-48h	81.43	1.14	80.88	1.17	12.97	0.68	31.63	1.11	7.98	3.64	1.53	0.45	69.73	3.14	73.01	3.13	43.07	0.76	124.67	1.75
Leaf-ABA-96h	61.97	0.78	74.77	1.00	12.23	0.75	33.89	1.39	3.65	0.95	2.83	0.65	27.87	0.45	64.04	1.49	44.81	1.07	130.36	1.97
Stem-ABA-24h	66.03	1.32	64.97	1.14	13.70	0.69	34.10	1.77	4.94	0.87	5.39	1.12	48.31	2.07	65.67	1.48	29.99	0.52	90.03	0.74
Stem-ABA-48h	51.21	0.86	61.05	1.30	12.95	0.54	30.36	1.29	6.34	1.20	2.79	0.25	38.13	1.80	17.33	0.48	35.92	0.46	84.65	0.53
Stem-ABA-96h	59.27	1.23	39.36	1.20	18.95	0.63	25.12	1.23	3.04	0.67	0.00	0.00	25.25	1.04	20.04	0.60	28.74	0.43	59.69	0.41
Leaf-GA-24h	61.95	0.69	58.88	0.82	18.67	0.86	39.26	1.79	3.47	1.93	1.93	0.65	29.40	1.01	14.66	0.38	36.64	0.85	84.58	1.04
Leaf-GA-48h	73.79	1.03	90.60	1.31	15.52	0.82	19.92	0.70	7.40	3.38	1.39	0.41	66.46	3.00	55.71	2.39	43.53	0.76	86.09	1.21
Leaf-GA-96h	92.27	1.17	87.65	1.18	13.66	0.84	18.86	0.77	6.60	1.71	0.35	0.08	56.95	0.92	65.34	1.52	55.06	1.31	84.39	1.28
Stem-GA-24h	67.90	1.35	65.99	1.15	17.34	0.87	32.02	1.66	5.43	0.96	2.27	0.47	38.42	1.65	60.13	1.35	39.45	0.68	100.42	0.82
Stem-GA-48h	59.43	0.99	80.02	1.70	12.29	0.52	19.61	0.83	3.38	0.64	3.14	0.28	35.50	1.68	29.36	0.81	18.68	0.24	37.51	0.23
Stem-GA-96h	72.64	1.51	57.65	1.76	21.73	0.72	16.70	0.82	5.72	1.27	0.00	0.00	42.41	1.74	23.29	0.70	39.39	0.58	41.69	0.28

C:control, ABA: Abscisic acid, GA:Gibberellic acid, Change fold: expression level of treatment/expression level of Control

B

		ABA(leaf)			ABA(stem)			GA(leaf)			GA(stem)		
		24h	48h	96	24h	48h	96	24h	48h	96	24h	48h	96
Ss	MGT1	-	-	-	-	-	-	-	-	-	-	-	-
So	MGT1	-	-	-	-	-	-	-	-	-	-	-	-
Ss	MGT2	-	-	-	-	-	-	-	-	-	-	-	-
So	MGT2	-	-	-	-	-	-	↑	↑	-	-	-	-
Ss	MGT3	-	-	-	↑	-	-	-	-	-	-	↓	-
So	MGT3	-	↑	↑	-	↑	-	-	-	↑	-	-	-
Ss	MGT4	-	↓	↓	-	-	-	-	↓	↓	-	-	↑
So	MGT4	-	↑	-	↑	↑	↑	↓	↑	↑	↑	↑	↑
Ss	MGT5	-	-	-	-	-	-	↓	-	-	-	-	-
So	MGT5	-	-	-	-	-	-	-	-	-	-	↑	↑
Ss	MGT6	-	-	-	-	-	-	-	-	-	-	-	-
So	MGT6	-	-	-	-	-	-	-	-	-	-	-	-
Ss	MGT7	-	-	-	-	-	-	-	-	-	-	-	-
So	MGT7	-	-	-	-	-	-	-	-	-	-	-	-
Ss	MGT8	-	-	-	-	-	-	-	-	-	-	-	-
So	MGT8	-	-	-	-	↓	↓	-	-	-	-	-	-
Ss	MGT9	-	↑	↓	↑	-	-	-	↑	-	-	-	-
So	MGT9	-	↑	-	-	↓	-	↓	↑	-	-	-	-
Ss	MGT10	-	-	-	-	↓	↓	-	-	-	-	↓	-
So	MGT10	-	-	-	-	-	↓	-	-	-	-	↓	↓

"-": no change, "↑": Up-regulate, "↓": Down-regulate
