# Supplementary material for: Comparative genomics revealed the gene evolution and functional divergence of magnesium transporter families in Saccharum
Source: BMC Genomics. 2019 Jan 24;20:83. doi: 10.1186/s12864-019-5437-3 (PMC6345045; doi:10.1186/s12864-019-5437-3)

|  |  | Simple name | | | | | | | | | | | | | | | | |
| --- | --- | --- | --- | --- | --- | --- | --- | --- | --- | --- | --- | --- | --- | --- | --- | --- | --- | --- |
| Gene name | Gene ID | lvs | stg1inf | stg2inf | anth | pist | sd5 | sd10 | emb | endo | ABA_root | ABA_shoot | H2O_root | H2O_shoot | PEG_root | PEG_shoot | NaOH_root | NaOH_shoot |
| SbMGT1 | Sb06g016880 | 0.00 | 0.00 | 0.00 | 0.59 | 0.00 | 0.78 | 2.17 | 0.00 | 3.48 | 0.01 | 0.00 | 0.79 | 0.00 | 0.42 | 0.00 | 0.58 | 0.00 |
| SbMGT2 | Sb07g025520 | 19.60 | 17.03 | 13.57 | 6.22 | 20.36 | 19.24 | 7.93 | 14.01 | 15.32 | 8.37 | 9.26 | 9.32 | 8.43 | 10.24 | 7.84 | 9.61 | 8.67 |
| SbMGT3 | Sb03g043270 | 39.01 | 25.51 | 45.60 | 26.28 | 33.66 | 24.20 | 13.71 | 26.31 | 23.82 | 10.22 | 8.57 | 10.26 | 8.16 | 12.77 | 8.72 | 10.72 | 7.73 |
| SbMGT4 | Sb06g021670 | 5.79 | 7.84 | 6.02 | 13.67 | 28.90 | 8.10 | 1.42 | 13.23 | 3.05 | 1.70 | 3.11 | 0.63 | 2.79 | 0.58 | 2.90 | 0.65 | 2.78 |
| SbMGT5 | Sb10g025720 | 14.66 | 11.21 | 11.67 | 20.74 | 13.13 | 13.70 | 5.74 | 9.68 | 8.08 | 10.32 | 14.28 | 12.57 | 16.16 | 12.18 | 12.04 | 13.06 | 14.49 |
| SbMGT6 | Sb01g008500 | 34.89 | 32.17 | 67.56 | 127.00 | 38.64 | 56.93 | 46.90 | 34.64 | 46.43 | 38.30 | 45.13 | 27.32 | 34.39 | 26.89 | 37.57 | 27.59 | 30.58 |
| SbMGT7 | Sb01g030170 | 26.50 | 23.11 | 38.91 | 40.65 | 39.90 | 34.29 | 20.79 | 18.51 | 22.42 | 24.45 | 23.49 | 24.03 | 22.16 | 28.26 | 22.48 | 25.57 | 21.02 |
| SbMGT8 | Sb01g047780 | 0.00 | 0.00 | 0.00 | 0.00 | 0.40 | 0.58 | 0.00 | 0.00 | 0.00 | 0.03 | 0.11 | 0.07 | 0.15 | 0.10 | 0.16 | 0.11 | 0.22 |
| SbMGT9 | Sb01g011810 | 9.43 | 7.30 | 9.68 | 43.59 | 15.33 | 19.99 | 3.23 | 20.13 | 2.28 | 1.84 | 40.53 | 0.89 | 15.93 | 0.72 | 31.55 | 0.76 | 16.48 |
| SbMGT10 | Sb03g043270 | 39.01 | 25.51 | 45.60 | 26.28 | 33.66 | 24.20 | 13.71 | 26.31 | 23.82 | 10.22 | 8.57 | 10.26 | 8.16 | 12.77 | 8.72 | 10.72 | 7.73 |

**RPKM**

**0**

**10**

**100**


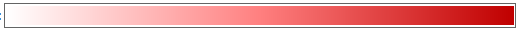

Supplement: Supplementary file 17 — The expression patterns of MGTs in Sorghumbicolor based on qTeller [57, 89]. Abbreviation: Lvs: Young leaves were harvested 20 days after sowing; stg1inf: Primodial inflorescences were harvested 10 days before flower emergence; stg2inf: Whole inflorescences were harvested at the time of flower emergence; anth: Whole anthers were harvested at the time anthesis; pist: Pistils were harvested at the time anthesis; sd5: Whole seeds were harvested 5 days after pollination; sd10: Whole seeds were harvested 10 days after pollination; emb: Developing embryos were harvested 25 days after pollination; endo: Developing endosperms were harvested 25 days after pollination. (DOC 58 kb) [file 12864_2019_5437_MOESM17_ESM.doc]
