# Supplementary material for: Comparative genomics revealed the gene evolution and functional divergence of magnesium transporter families in Saccharum
Source: BMC Genomics. 2019 Jan 24;20:83. doi: 10.1186/s12864-019-5437-3 (PMC6345045; doi:10.1186/s12864-019-5437-3)
Supplement: Supplementary file 13 [file 12864_2019_5437_MOESM11_ESM.doc]

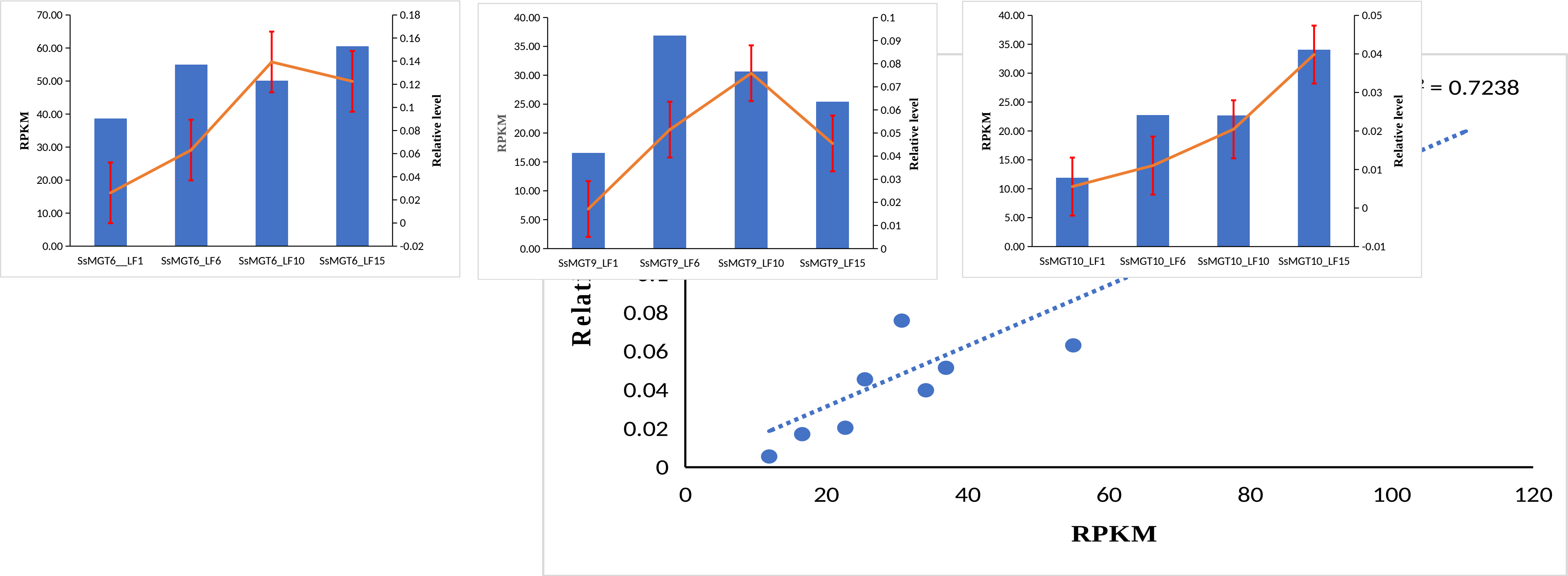

RPKM
Relative level
SsMGT6__LF1
SsMGT6_LF6
SsMGT6_LF10
SsMGT6_LF15
SsMGT9_LF1
SsMGT9_LF6
SsMGT9_LF10
SsMGT9_LF15
SsMGT10_LF1
SsMGT10_LF6
SsMGT10_LF10
SsMGT10_LF15
= 0.7238
Relat
RPKM
